# Multiscale network reorganization during the multidimensional recollection of naturalistic events

**DOI:** 10.1007/s00429-026-03184-8

**Published:** 2026-08-27

**Authors:** Federica Procida, Maria Giulia Tullo, Matteo Frisoni, Annalisa Tosoni, Mauro Gianni Perrucci, Antonello Baldassarre, Carlo Sestieri

**Affiliations:** 1 Department of Neuroscience, Imaging and Clinical Sciences, Institute of Advanced Biomedical Technologies, University G. d’Annunzio of Chieti-Pescara, Via dei Vestini 31, 66100 Chieti, Italy; 2 ITAB Institute for Advanced Biomedical Technologies, University G. d’Annunzio of Chieti- Pescara, Via dei Vestini 31, 66100 Chieti, Italy; 3 Department of Psychology, ‘Sapienza’ University of Rome, Via dei Marsi, 78, 00185 Rome, Italy; 4 Department of Psychology (DiPSI), University G. d’Annunzio of Chieti-Pescara, Via dei Vestini 31, 66100 Chieti, Italy; 5 Research Center, UdA-TechLab, University “G. d’Annunzio” of Chieti-Pescara, Chieti, Italy

**Keywords:** Cued recollection, Naturalistic stimuli, Graph theory, Default mode network, Hippocampus

## Abstract

The recollection of complex events is multidimensional. Recent work has gathered evidence for varying degrees of functional specialization for different memory dimensions across the main cortical networks involved in cued recollection. However, how functional brain network topology reorganizes to support the retrieval of different types of information remains largely unexplored. Here, we applied graph theoretical analysis to task-related fMRI functional connectivity data from 22 participants engaged in cued recollection of specific details from naturalistic movie scenes. A beta series correlation approach was used to identify the pattern of whole-brain regional co-activation for cued recollection in general, and separately for each memory dimension of interest (object and character details, spatial layouts, temporal sequences, and verbal dialogues). Subsequently, using graph theory, we investigated the association between indices of brain network topology and cued recollection performance, as well as differences in functional brain network architecture across memory dimensions at the multiscale level. Successful recollection was associated with higher global efficiency and lower modularity at the whole-network level, as well as with lower system segregation within the Frontoparietal control network. Crucially, retrieval conditions placing greater demands on relational integration (i.e., temporal order judgments), compared to those emphasizing single-item details, were associated with more integration in the Default Mode network and increased degree centrality, efficiency, and reduced path length in the right hippocampus. Together, these findings reinforce the idea that successful retrieval requires system integration, especially when accessing complex relational representations like temporal sequences.

**Supplementary Information:**

The online version contains supplementary material available at https://doi.org/10.1007/s00429-026-03184-8.

## Introduction

Memories of complex events contain a large amount of precise, high-fidelity episodic details such as character/object physical features (e.g., the color of a hat), location (i.e. how the object/details in an event are arranged), the temporal sequence of actions, but also verbatim-like “record of dialogues” (Frisoni et al. 2023; Grilli and Sheldon 2022). Previous research has identified two main large-scale neural networks involved in episodic retrieval: the Frontoparietal Control Network (FPCN) and the Default Mode Network (DMN) (Sestieri 2011, 2017, Kuhl and Chun 2014; Spreng 2010; Fornito et al. 2012; King et al. 2015; Kim 2020). The FPCN exhibits a broad involvement in memory-related tasks, independent of their specific content (Cabeza et al. 2008; Ranganath and Ritchey 2012; Rugg and Vilberg 2013), whereas DMN supports introspective processes that allow individuals to reconstruct and mentally project themselves into past experiences (Andrews-Hanna 2012; Raichle 2015). Accordingly, in a previous fMRI study comparing cued recollection of different memory dimensions (Procida et al. 2025), we observed a higher degree of functional specialization within the DMN for the processing of different types of mnemonic information compared to the general activation pattern observed in the FPCN. We further demonstrated that such division of labor observed in the DMN is generally beneficial for recollection performance. Consistent with prior work using different retrieval paradigms (Kwok and Macaluso 2015; Park et al. 2023), these results underscore how specialized sub-regions within the DMN network support distinct dimensions of episodic retrieval.

However, one potential limitation of the ecological approach employed in our previous study concerns the potential heterogeneity of memory dimensions, in terms of the amount of retrieved information needed to judge the written statements that represented the memory cues. For example, statements from the temporal dimension often tested memory for the order of two events within a specific scene, requiring the relational integration of multiple memory traces into a unified representation. In contrast to such high demand on relational memory, the object/character dimension typically required the precise evaluation of statements regarding unique objects/characters, emphasizing item memory rather than memory for the spatial-temporal context in which items occurred. We previously acknowledged this as a potential limitation for the interpretation of topographic differences across conditions in our previous work. However, we also reasoned that this feature might indeed be leveraged to study whether a recollection process that requires different levels of integration (e.g., temporal dimension), compared to retrieving information about single items (e.g., object/details), is associated with variation in metrics of brain activity that can capture network integration/segregation at multiple levels (i.e., local/global) of the human connectome.

A powerful approach to address this question is to analyze functional connectivity during task execution using multivariate analytic methods, such as graph theory. Previous studies linking graph measures to task performance (Bassett et al. 2011; Braun et al. 2015; Cao et al. 2014) have shown that successful memory retrieval involves a reconfiguration of the network topology (Geib et al. 2017a, b; King et al. 2015; Schedlbauer et al. 2014). For example, Schedlbauer et al. (2014) found that many of the brain’s shortest topological paths traverse the hippocampus during successful recollection, while King et al. (2015) reported increased interconnectivity among retrieval-related regions predicting memory accuracy. Complementary evidence from Westphal et al. (2017) suggests that reduced network modularity, reflecting stronger integration between the FPCN and DMN, supports better memory performance. In a different line of research, Geib and colleagues (2017a, b) demonstrated that the hippocampus enhances its communication efficiency and centrality during successful remembering, in line with the idea that the hippocampus contributes to episodic memory through its dynamic communication with spatially distributed cortical regions (Geib et al. 2017a; Jeong et al. 2015; Mišić et al. 2014; Watrous et al. 2015).

To summarize, the existing literature highlights the role of the hippocampus and other retrieval-related regions in orchestrating the network reconfiguration necessary for successful memory performance, which is characterized by a transition toward lower modularity (i.e., greater integration across network communities) and higher global efficiency (i.e., more efficient communication across the network). Notably, although few studies have compared different memory dimensions (Schedlbauer et al. 2014; Cooper and Ritchey 2019), to our knowledge, no study has consistently investigated the issue within the recollection of complex audiovisual narratives. In the present fMRI study, we used our existing database (Procida et al. 2025) and applied a network neuroscience framework to investigate how large-scale brain architecture supports cued recollection, focusing on four memory dimensions (object and character details, spatial layouts, temporal sequences, and verbal dialogues).

Based on the existing literature and the distinct cognitive demands required for item-based versus relational event retrieval, we formulated two main hypotheses. First, extending prior work showing that reduced network modularity supports better memory performance (Westphal et al. 2017), we hypothesized that individual differences in cued recollection performance, regardless of the specific dimension, would be associated with a more globally integrated brain architecture (i.e., lower modularity), reflecting more efficient communication across distributed regions of the retrieval network. This relationship was tested at three levels: at the level of the whole-brain connectome, focusing on indices of global efficiency and modularity; at the network level, focusing on system segregation/integration of the two main resting state networks (the DMN and the FPCN) that have been most strongly associated with cued recollection (Sestieri 2011, 2017, Kuhl and Chun 2014; Spreng 2010; Fornito et al. 2012; King et al. 2015; Kim 2020), and, finally, at the nodal level, focusing on the centrality of the hippocampus (Schedlbauer et al. 2014; Geib and colleagues 2017a, b). Second, we hypothesized indices of network integration to differ between memory dimensions, especially between the temporal one, which requires integrating multiple event elements into a unified representation, and the one referring to object and character details, which emphasizes the retrieval of discrete, single-item information. Again, we tested for a significant difference in metrics referring to the whole-network, network-community, and node level, expecting a potential effect on the whole connectome, but also on specific networks (i.e., DMN, FPCN) and nodes (i.e., the hippocampus) that are considered hubs for coordinating distributed memory representations (Eichenbaum 2017; McCormick et al. 2018).

## Methods

### Participants

Detailed information regarding the study participants can be found in our previously published paper (Procida et al. 2025). Briefly, twenty-four right-handed volunteers participated in the study. Two participants were excluded from analyses based on an a priori criterion: accuracy falling more than 2 SD below the group mean on at least one dimension. Both excluded participants showed multiple values below this threshold. Thus, analyses were performed on 22 participants (12 females; mean age: 25.4; sd: 2.4). Participants provided informed consent before the experiment according to guidelines set by the Human Studies Committee of G. d’Annunzio University of Chieti (May 30, 2019). The study included an encoding session (duration: 90 min, fMRI data not used in the present manuscript) and a retrieval session (duration: 90 min). The two sessions were separated by a ~ 24 h interval.

### Study material

During the encoding session, participants viewed a chaptered version of the first 58 min of the *Sherlock* episode *A Study in Pink* (Season 1, Episode 1; total duration 87 min 30 s; BBC 2010), dubbed in Italian and divided into 31 discrete chapters or scenes (Chen 2017). Each chapter began with an 8-second black screen displaying a numerical and descriptive title (e.g., “#1 Therapy” or “#16 Sherlock and Watson in a taxi”) to facilitate the identification of the corresponding scene during the subsequent retrieval session. The segmentation and labeling of scenes were based on a pilot study in which an independent group of 20 participants was asked to segment the episode into distinct narrative events following the *Event Horizon Model* framework (Radvansky and Zacks 2017; see Frisoni et al. 2021 for details). Of the 31 scenes presented during encoding, only 25 were included in the retrieval phase, as scenes that were too brief to generate enough memory-related sentences were excluded. The first scene was used for the practice session. Four memory dimensions were defined, capturing the main types of information that people naturally retrieve when remembering a complex event. When individuals recall a movie scene or an everyday episode, they typically reconstruct what was present, where elements were located, how the event unfolded over time, and what was said by the main characters. At a broader narrative level, episodic retrieval involves integrating these sources of information into a coherent representation, combining memory for “what” occurred with memory for “where” and “when” it took place (Tulving 1972, 1983; Cheke and Clayton 2013). Beyond this spatiotemporal scaffolding, episodic representations preserve fine‑grained perceptual and contextual details, including the physical features of characters and objects, the spatial configuration of the scene, the temporal ordering of actions, and the content of dialogues (Grilli and Sheldon 2022). Notably, although the statements belonging to the verbal dimension did not reproduce dialogue verbatim, they nonetheless retained the lexical content and propositional structure of characters’ utterances. Consistent with the three‑level model of text representation (Van Dijk and Kintsch 1983; Glenberg et al. 1987), memory for what was said, even when paraphrased, constitutes a well‑established and distinct dimension of episodic memory (Rubin and Umanath 2015).

A total of 396 sentences (198 true and 198 false) were used to probe memory for events across the movie (see Supplementary Material). Several of these sentences had already been employed in a recent behavioral study (Frisoni et al. 2023), in which the full movie was shown to a different participant sample. After excluding 12 sentences referring to the first scene, used solely for the practice phase, the remaining 384 sentences covered 24 movie scenes: 288 served as standard trials, and 96 as catch trials (see the “Procedure” section below). Specifically, 16 sentences were selected for each of the 24 retrieval scenes, 4 sentences for each of the 4 dimensions of interest: dialogue or verbal information (e.g., “The widow says the man was undoubtedly murdered”), object or character details (e.g., “Sergeant Donovan wore red glasses”), temporal order between events (e.g., “The boys walk together before the victim walks away”), and spatial layout (e.g., “The suitcase was on the chair”). The average sentence length was 9.3 ± 1.4 words (mean ± SD) or 57.5 ± 6.0 characters (including spaces). For more details on the procedures, see Procida et al. (2025).

The statements differed in their linguistic properties across dimensions. For example, the average sentence length was 8.8 ± 1.3 words for object/character sentences, 9.2 ± 1.2 for spatial, 9.7 ± 1.4 for temporal, and 9.7 ± 1.3 for verbal sentences (see Supplementary Material). However, the variability was intrinsic to the current manipulation: to direct retrieval toward a specific memory dimension, sentences necessarily had to be structured in ways that selectively cue the type of information required. A morphosyntactic analysis confirmed that each dimension carries the linguistic signature expected by its cognitive definition, such as object/character sentences ranked highest in adjective count (M = 0.96), spatial in noun count (M = 3.67), temporal in adverb count (M = 0.84), and verbal in verb count (M = 2.49). (Detailed analyses are reported in the Supplementary Material).

### Procedure

During the encoding session, participants viewed the chaptered episode across 15 encoding runs, each of which concluded with a 1-minute resting-state period. The encoding session was preceded and followed by two resting-state runs.

**Fig. 1:**
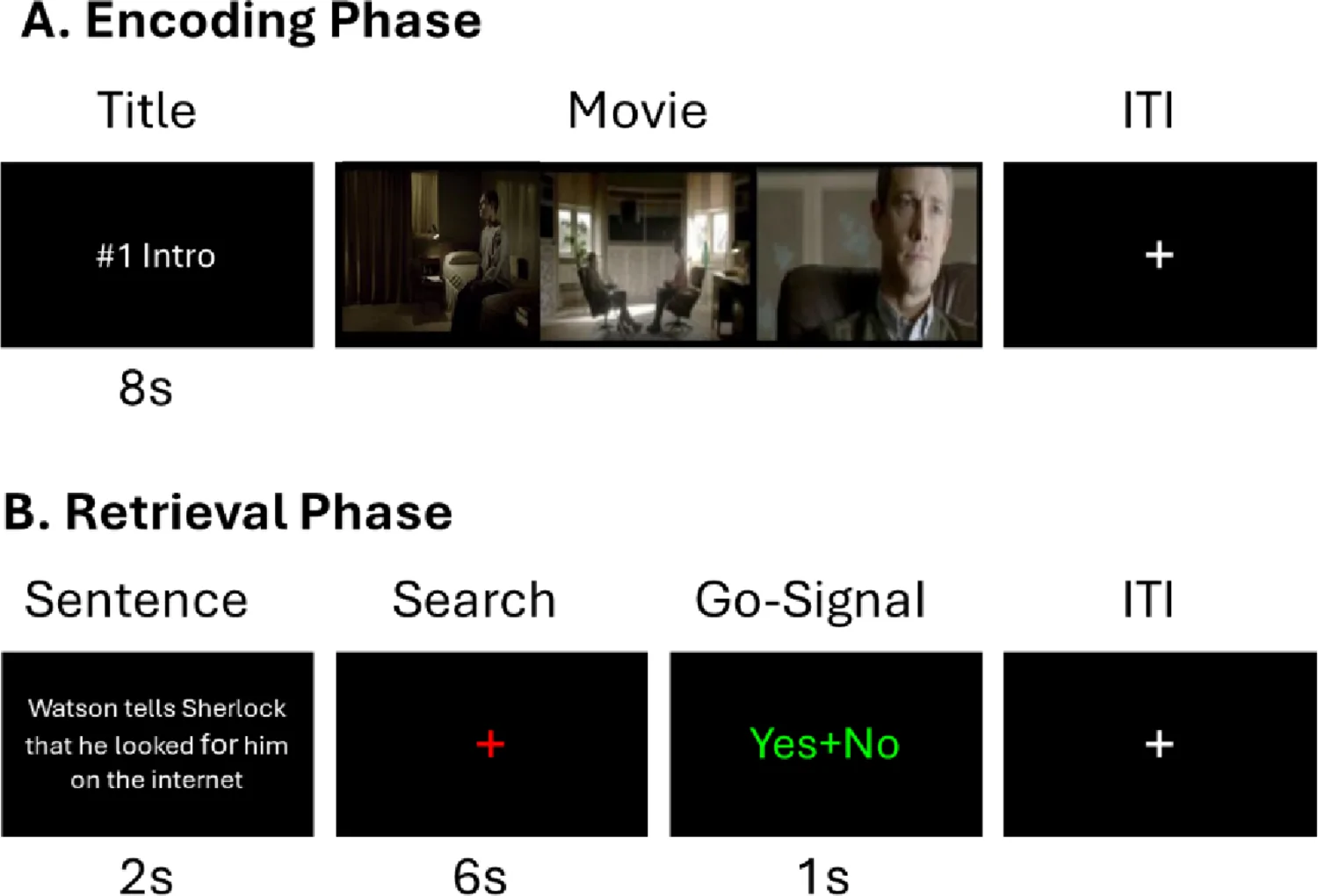
The figure illustrates the structure of the experimental paradigm. **A** In the encoding session, participants were presented with the first ∼60 min of an episode of the TV series “Sherlock”. An 8-second black screen containing a specific scene title was inserted into the original movie and appeared on the screen at the beginning of each scene. **B** In the retrieval session, participants were cued with a scene title (the same one seen during movie viewing), followed by a group of sentences addressing detailed memory for that scene. Each sentence lasted for 2 s and was followed by a memory search phase of a fixed duration (6 s), a response phase (Go-Signal, 1 s), and an ITI of variable duration. During catch trials, the ITI immediately followed the sentence, and the participant was told not to start the memory search. (adapted from Procida et al. 2025)

The retrieval session consisted of 12 runs, each including statements referring to two consecutive chaptered scenes presented in the same chronological order as in the movie. Each run began with an 8-second presentation of the relevant scene title, followed by 16 trials about that scene (12 standard and 4 catch trials; see Fig. 1A). The trial order was randomized within each scene. After each set of trials, a new title was presented to indicate the transition to the subsequent scene. The correspondence of scene titles between the encoding and retrieval phases was intended to facilitate accurate scene identification and support event-specific retrieval.

In standard trials, a sentence was presented centrally for 2 s, followed by a memory search period of 6 s. During this period, a black screen with a red fixation cross was displayed, and participants were instructed to retrieve the episodic information necessary to judge the truthfulness of the sentence (see Fig. 1B). The term *memory search* is used to denote the coordinated set of cognitive operations underlying goal-directed episodic retrieval and subsequent evaluation of retrieved content (Moscovitch and Winocur 1995; Mecklinger 2010). Participants were instructed to withhold their response until the onset of a go signal, at which point they indicated whether the sentence was true or false and simultaneously reported their confidence about the response. There were two button boxes, one for each hand, each with two buttons. External buttons always indicated high confidence, whereas internal buttons indicated low confidence judgments, but the association between the hand (left/right) and the response (yes/no) was randomly assigned on every trial and known to subjects only at the onset of the go-signal. This approach was critical in ensuring that motor intentions did not affect the memory search phase of the trial. The go signal was displayed for 1 s, followed by a white fixation cross during a variable intertrial interval (3.5–5.5 s). Catch trials were included to isolate neural responses associated with sentence reading from those related to memory search (Sestieri et al. 2010, 2011). In these trials, the sentence was immediately followed by the intertrial interval, and participants were instructed to refrain from retrieving any related information (see below).

### Apparatus and data acquisition

Imaging data were acquired using a Philips Achieva SmartPath to dStream 3 Tesla MR scanner (Philips Medical System, Best, The Netherlands) located at the Institute of Advanced Biomedical Technologies (ITAB) in Chieti, Italy. Head motion was minimized using foam padding. Visual stimuli were projected on a screen located at the head of the magnet bore via an LCD projector and viewed through a mirror attached to the head coil. Auditory stimuli in the encoding phase were delivered using a pneumatic headset designed to minimize interference from scanner noise. E-Prime 2.0 (Psychology Software Tools) was used for presenting stimuli, synchronizing stimulus presentation with MRI data acquisition via scanner trigger, and collecting responses. Manual responses were recorded using a Cedrus Lumina LU400 fiber optic Response Pad.

Functional T2*-weighted images were collected using a sensitivity-encoding 32-channel brain coil and gradient‐echo EPI sequences using BOLD contrast over the whole brain (Kwong et al. 1992), with the following parameters: 48 transverse slices, TR = 1000 ms, TE = 30 ms, flip angle = 65°, voxel size: 3 × 3 × 3 mm, FOV = 240 × 240 × 144 mm. Structural images were obtained using a sagittal magnetization-prepared T1-weighted Turbo Field Echo sequence in an interleaved order with the following parameters: 190 slices, TR = 8.1 ms, TE = 3.7 ms, flip angle: 8°, voxel size: 1 × 1 × 1 mm, FOV = 256 × 190 mm.

### Behavioral data analysis

We computed general accuracy, combining hits and correct rejections. A one-way repeated-measures ANOVA with dimension as a factor, followed by Bonferroni-corrected post hoc tests, was used to test for differences in memory accuracy between memory dimensions (Procida et al. 2025).

### Data preprocessing

Data pre-processing was performed using SPM12 (Wellcome Center for Human Neuroimaging, https://www.fil.ion.ucl.ac.uk/spm) and the FMRIB Software Library (FSL) (Jenkinson et al. 2012; Smith et al. 2004). Skull stripping of anatomical images was performed with FSL. Functional images were realigned to be correct for head movements, and slice-acquisition delays were corrected using the middle slice as a reference. Images were then normalized to the Montreal Neurological Institute (MNI) template and spatially smoothed using an isotropic Gaussian kernel of 4 mm FWHM. Nuisance regressors included six motion-related parameters (three for translations and three for rotations; range: 0.081–0.202 mm; median motion: 0.116 mm) and framewise displacement (FD), a subject-specific time-series index representing the overall movement estimate over time (Power et al. 2012). FD was computed as the sum of the absolute temporal derivatives of the six motion-related parameters.

### General linear model and functional brain network construction

To create matrices of inter-regional context-dependent functional connectivity, we employed a β‑series correlation approach (Rissman et al. 2004), which estimates functional coupling between two regions as the correlation between their trial‑wise beta estimates for a given condition (Fornito et al. 2011; Schedlbauer et al. 2014). This approach requires the computation of a general linear model (GLM) specifying individual regressors for each trial of interest (memory search), whereas the title, the sentence reading (across conditions), and the three response-related regressors (left, right, and no responses) were modeled across trials. Importantly, the use of catch trials allowed the estimation of Blood-Oxygenation Level‐Dependent (BOLD) activity specific to sentence reading, which could subsequently be linearly subtracted from the overall activity measured in standard trials (Shulman et al. 1999; Ollinger et al. 2001). This occurs because a single-sentence reading predictor for catch and standard trials was included in the GLM. Accordingly, the brief presentation time (2 s) of the sentences was chosen to minimize the possibility that participants started the memory search when reading statements in catch trials. We acknowledge that we cannot guarantee that participants refrained from covertly initiating a memory search on catch trials. However, any unwanted retrieval-related activity that unintentionally occurred on catch trials would be explained by the sentence-reading predictor. As a result, the estimated memory-search activity reflects a conservative measure of the neural processes engaged during deliberate retrieval.

Each regressor specified the onset of the trial convolved with the canonical hemodynamic response function (HRF). As a result, we obtained 288 β values for each brain voxel reflecting the hemodynamic response evoked by memory search in each trial, which could be sorted by recollection accuracy (correct, incorrect) and retrieval dimension (object and character details, spatial layouts, temporal sequences, and verbal dialogues). All subsequent analyses were performed on correct trials only (an average of 54 ± 6 trials (SD) per dimension). We also conducted an additional analysis testing for differences between correct and incorrect trials, regardless of memory dimensions (Supplementary Material).

The next step involved the definition of ROIs covering the whole brain. The brain was first parcellated into 100 discrete anatomical regions of interest (50 ROIs in each hemisphere), defined in accordance with the Schaefer atlas (Schaefer et al. 2018). The (left and right) hippocampus was extracted from the Harvard-Oxford Cortical Structural Atlas (RRID: SCR_001476) (2025). The beta series of voxels belonging to a ROI were subsequently averaged, and the Pearson correlation coefficient between all ROI’s beta series was computed, creating a correlation, or adjacency, matrix of all pairwise combinations (102 × 102), describing the strength of the functional relationship between two ROIs. We obtained both a general matrix considering all trials independent of dimension and four dimension-specific matrices.

Brain connectivity can be represented as a graph, which consists of a set of nodes together with pairwise connections between those nodes (edges). In these functional brain networks, each node represents a discrete brain region, and edges represent measured correlations between pairs of nodes. Importantly, consistent with previous work using a similar beta series correlation approach (Rubinov and Sporns 2010; Geib et al. 2017a, b), the adjacency matrices were not thresholded (i.e., weak connections were not removed) and each matrix constituted an undirected, weighted graph. For each graph theory metric (Fig. 3A, B), the diagonal and negative values set to zero, because of the present ambiguity regarding the meaning of negative correlations (anticorrelated links) (see Telesford et al. (2011) and Cao et al. (2014).

### Graph theory measures

#### Global efficiency

Global efficiency was used to quantify the network’s capacity for rapid information integration across distant regions. In weighted functional networks, the ‘distance’ between two regions refers to their functional communication cost and is computed as the length of the shortest path connecting them, where stronger functional connections correspond to shorter effective distances. In this context, *N* denotes the total number of nodes in the network. For each node (*n*), efficiency is calculated as the average inverse distance to all other regions, normalized by the number of possible connections (*n-*1). This normalization ensures comparability across nodes and networks of different sizes. Network-wide global efficiency is then obtained by averaging nodal efficiency values. Global efficiency is formally defined as (Latora and Marchiori 2001; Rubinov and Sporns 2010):

$$\:{E}^{w}=\:\frac{1}{n}{\sum\:}_{i\in\:N}\frac{{\sum\:}_{j\in\:N,j\ne\:i\:}\left({d}_{ij}^{w}\right)-1}{n-1}$$

where $\:{d}_{ij}^{w\:\:}$ is the shortest weighted path length between two nodes in the network after taking into account the strength of each connection. For modular connectivity analyses, e_i_ⱼ refers to the total weight of edges linking module i to module j, normalized by the number of possible inter-module connections. For each node, efficiency is calculated as the average inverse distance to all other nodes; this value is then averaged across all nodes to yield a single network-level metric. The network-wide global efficiency is computed as an average of all nodal-wise global efficiency values. The resulting value ranges between 0 and 1, with higher values indicating more efficient global information transfer.

#### Modularity

Modularity, an index of the degree to which nodes in a complex system are organized into discrete communities, has emerged as an important construct for characterizing large-scale brain network organization and connectivity dynamics (Westphal et al. 2017). To the extent that lower modularity facilitates internetwork communication, it may reflect a shift toward more globally integrative processing.

Modularity is computed using an optimization algorithm that identifies subsets of nodes that are more densely interconnected with each other than with the rest of the network (Blondel et al. 2008; Newman 2006; Newman and Girvan 2004). The degree to which a network can be partitioned into such nonoverlapping modules is quantified by the modularity statistic Q. Higher Q values indicate a more segregated network architecture, characterized by clearly delineated functional communities, whereas lower Q values reflect a more integrated network organization with less distinct modular boundaries. The modularity value for a given partition of the functional brain network is computed as follows:

$$\:Q=\:\sum\:_{i=1}^{k}\left[\frac{{e}_{ij}}{M}-{\left(\frac{{a}_{i}}{M}\right)}^{2}\right]$$

where $\:{e}_{ij\:}$ is a measure of within-module connections in module * i*, $\:{a}_{i\:}$ is the total degree (summed strength of all connections) of module * i*, * M* is the total degree of the entire network, and * k* is the total number of modules. Higher * Q*values indicate a more segregated network architecture with clearly delineated functional communities, whereas lower * Q* values reflect a more integrated organisation with less distinct modular boundaries.

Modularity algorithms are designed to maximize $\:Q\:$ by identifying non-overlapping node subsets that jointly maximise within-module and minimise between-module connectivity. In the current study, the weighted undirected Louvain algorithm (Blondel et al. 2008) was applied separately at the individual and group levels. At the individual level, the algorithm was run 10 times per participant, as modular partitions were highly stable across repetitions and additional iterations did not alter the resulting community structure (cf. Geib et al. 2017a, b; Deng et al. 2021). At the group level, the algorithm was run 1,000 times on the average connectivity matrix across participants, to ensure convergence on a robust consensus partition. In both cases, the partition yielding the highest * Q* value was retained for further analyses. Following prior work (e.g., Geib et al. 2017a, b), the algorithm was run a limited number of times at the individual‑subject level because modular partitions were highly stable across repetitions, and additional iterations did not change the resulting community structure. In contrast, the group‑level modular assignment was derived from the average connectivity matrix and therefore required a larger number of iterations to ensure convergence on a robust consensus partition. This procedure is consistent with standard practice in consensus community detection.

#### System segregation

System segregation indexes the degree to which major functional systems preserve strong internal cohesion relative to their connectivity with other systems (Wig 2017). While the modularity is calculated as the fraction of edges that fall within the given communities of a network compared to the expected fraction if the connections were distributed at random across the network [Q (Newman 2006)], segregation is calculated as the difference in mean within- versus between-community connections, relative to the mean within-community connections of a network (Chan et al. 2014; Wig 2017). Furthermore, while networks with higher modularity (and/or higher segregation) have greater separation between their communities, lower system segregation reflects increased inter-system communication and is often interpreted as a shift toward more integrated processing.

$$\:Segregation=\frac{within\:connections-\:between\:connections}{within\:connections}$$

#### Degree centrality

Degree centrality (DC) captures the relevance of individual nodes within the network by quantifying their overall connectivity strength, computed as the sum of the weights of all their direct connections (Bullmore and Sporns 2009). Nodes with high degree centrality typically engage in direct interactions with numerous other nodes, positioning them as key hubs in the network’s information flow.

$$\:{DC}_{i}^{w}=\:{\sum\:}_{j}^{N}{W}_{ij}$$

where W_ij_ represents the weighted adjacency matrix in which W_ij_ > 0 if node $\:i\:$ is connected to node * j* and the value given to the link is the weight of the connection. Nodes exhibiting high degree centrality engage directly with numerous other nodes in the network, making them potentially influential in shaping overall network dynamics and pivotal in mediating the flow of information or resources.

#### Characteristic path length

Path length indexes the efficiency of information transfer between pairs of nodes and is defined as the average shortest path connecting nodes within a network. A path consists of a sequence of distinct nodes and edges, and in brain networks these paths represent potential routes for information flow between regions, with shorter paths indicating a greater capacity for global integration (Bullmore and Sporns 2009; Rubinov and Sporns 2010). At the nodal level, path length is defined as the mean shortest distance between a given node *i* and all other nodes in the network (Watts and Strogatz 1998). In weighted functional brain networks, stronger connections—reflected by higher correlation strengths—are treated as shorter distances, whereas weaker connections correspond to greater distances (Rubinov and Sporns 2010). The path length between nodes $\:i\:$ and $\:j\:$ is defined as the sum of these inverse edge weights along the connecting path, such that the weighted shortest path L is the minimum of these sums across all possible routes *i* and *j*. Network-level weighted characteristic path length $\:{L}_{net}^{w}$ is then computed by evaluating the shortest path lengths between all node pairs in the network (Geib et al. 2017a, b):

$$\:{L}_{net}^{w}=\frac{1}{\frac{1}{N(N-1)}{\sum\:}_{i=1}^{N}{\sum\:}_{j\ne\:i}^{N}\frac{1}{{L}_{ij}^{w}}}$$

where $\:N\:$ is the total number of nodes in the network. This formulation, based on the harmonic mean, ensures robustness to disconnected node pairs, which would otherwise produce undefined path lengths. Shorter path lengths indicate that information spreads rapidly across the network, facilitating efficient distributed and parallel information processing and reflecting greater global integration.

#### Nodal global efficiency

Global efficiency at a node represents a measure of that node’s centrality within the network, characterizing the degree of global connectedness of each region. A node’s global efficiency is defined as the average of the inverse characteristic path length between that node and all other nodes in the network (Rubinov and Sporns 2010), such that higher values correspond to shorter path lengths and more efficient communication. In the expression below, $\:{d}_{ij}$ refers to the shortest path length connecting node $\:i\:$ and node * j*, and * n* corresponds to the total number of nodes in the network:

$$\:{E}_{i}=\frac{1}{n-1}\sum\:_{j\ne\:i}^{N}\frac{1}{{d}_{ij}}$$

where $\:i\:$ and $\:j\:$ index the nodes of the network, $\:N\:$ denotes the full set of nodes, and $\:n-1\:$ is the number of nodes reachable from $\:i\:$. For each node pair $\:(\:\mathrm{i}\:,\mathrm{j}\:)$, the shortest path distance $\:{d}_{ij}\:$ is defined as the minimum sum of edge lengths along any connecting path, where each edge length corresponds to the reciprocal of its weight ($\:1/{w}_{ij}$). When no path existed between two nodes, $\:{d}_{ij}$ was set to infinity, yielding an efficiency contribution of zero. Nodal efficiency thus provides a local measure of how efficiently information originating from a given node can be transmitted throughout the network.

#### Statistical analyses of graph theory measures

Graph-theoretic metrics were computed using the Brain Connectivity Toolbox (RRID: SCR_004841; Rubinov and Sporns 2010). Then, correlations were used to assess associations between each metric and behavioral performance. The analysis of the correlation between graph-theoretical metrics and behavioral performance was computed on a concatenated matrix of the four memory dimensions (details, spatial, temporal, verbal). To measure the relationship between behavioral performance and each index, we performed a series of correlation analyses for a total of 10 correlations organized across three analytical scales: 2 at the whole‑network level (global efficiency and modularity), 2 at the network community level (FPCN and DMN segregation), and 6 at the node wise level (path length, degree centrality, and nodal efficiency for left and right hippocampus). Each analytical level (whole-brain metrics, network-level indices, node-level hippocampal measures, and dimension-based comparisons) was treated as a separate family of tests, consistent with their conceptual and hypothesis-driven distinction in the analytical framework. Within each family, we applied the Benjamini–Hochberg False Discovery Rate (FDR) correction to control for multiple comparisons. Correlation analyses were performed in Jamovi (v2.6.44).

To examine differences across memory dimensions for each graph-theoretical measure while controlling for behavioral accuracy, which varied systematically across dimensions, we fitted a separate linear mixed-effects model (LMM) for each metric. Each model included dimension (DE, SP, TE, VE) as a categorical fixed factor, accuracy (mean-centered) as a continuous covariate whose values were condition-specific, modeled with a single common slope across dimensions, and by-subject random intercepts. This framework allowed us to estimate the effect of dimension while simultaneously accounting for accuracy and for the correlation between repeated measures within subjects, thereby providing a more stringent test of whether the observed dimension effects reflected mnemonic processing rather than systematic differences in retrieval performance across conditions. All models were estimated by maximum likelihood to enable likelihood-ratio testing. The overall effect of dimension for each metric was evaluated by comparing the full model with a reduced model that omitted the dimension factor, using a likelihood-ratio test (LRT) with a χ² approximation, where the degrees of freedom corresponded to the difference in the number of estimated parameters between the two models.

When the dimension effect was significant, pairwise comparisons among all six pairs of dimensions were carried out within the same mixed-effects framework. For each contrast, one dimension was set as the reference level so that the corresponding fixed-effect coefficient estimated the accuracy-adjusted difference between the two dimensions. Because each pairwise difference corresponded to a single model parameter, it was tested directly with a Wald *z* test. By successively changing the reference category and re-fitting the model, all six pairwise differences were obtained while continuing to control for accuracy and to account for the within-subject correlation. The resulting *p*-values were corrected for multiple comparisons using the Benjamini–Hochberg false discovery rate (FDR) procedure (α = 0.05, k = 6).

When the dimension effect was not significant, we restricted the analysis to a single, a priori contrast of interest, comparing the TE and DE dimensions, computed with the same re-parameterization and Wald *z*-test described above. The corresponding *p*-values were likewise corrected for multiple comparisons using the FDR procedure. The rationale was that these two dimensions rely on partly distinct memory processes, with the temporal dimension (TE) drawing on the integration of separate event elements into a coherent representation and the detail dimension (DE) relying instead on the retrieval of individual, item-specific information. The LMM analyses were implemented in Python (version 3.13.5) using the statsmodels library (Seabold and Perktold 2010). The effect of condition on different indices of graph theory was also tested with classical repeated-measures ANOVAs with dimension as a factor (See supplementary materials).

## Results

### Behavioral results

Overall task accuracy was 0.77 ± 0.06 (mean ± SD) with the following levels of performance across the 4 conditions: 0.79 ± 0.06 for the details, 0.79 ± 0.07 for the spatial, 0.74 ± 0.08 for the temporal, and 0.76 ± 0.08 for the verbal dimension (Fig. 1C in Procida et al. 2025). The ANOVA indicated a significant effect of dimension (*p* < 0.001), which was explained by relatively less accurate performance in the temporal dimension compared to details (*p* < 0.01) and spatial dimension (*p* < 0.01). To control the possibility that differences in behavioral performance explain differences in BOLD activity between dimensions, accuracy was used as a covariate in subsequent analyses examining dimension-specific indices of BOLD activity.

### Associations between graph metrics and behavioral performance

At the whole‑network level, cued recollection accuracy was associated with higher global integration and reduced modular network configurations, as shown by a positive correlation with global efficiency (*r* = 0.43, *p* = 0.043, p_fdr = 0.043) and negative correlations with modularity (*r* = − 0.39, *p* = 0.037, p_fdr = 0.043), respectively.

Furthermore, at the network‑community level, system segregation within the Frontoparietal control network (FPCN) was also negatively correlated with recollection accuracy (*r* = − 0.459, *p* = 0.032; p_fdr = 0.041), suggesting that less system segregation was associated with a greater memory performance. No significant correlation was observed for the DMN, nor for the remaining networks that served as a control for network specificity (all *p* > 0.05).

**Fig. 2:**
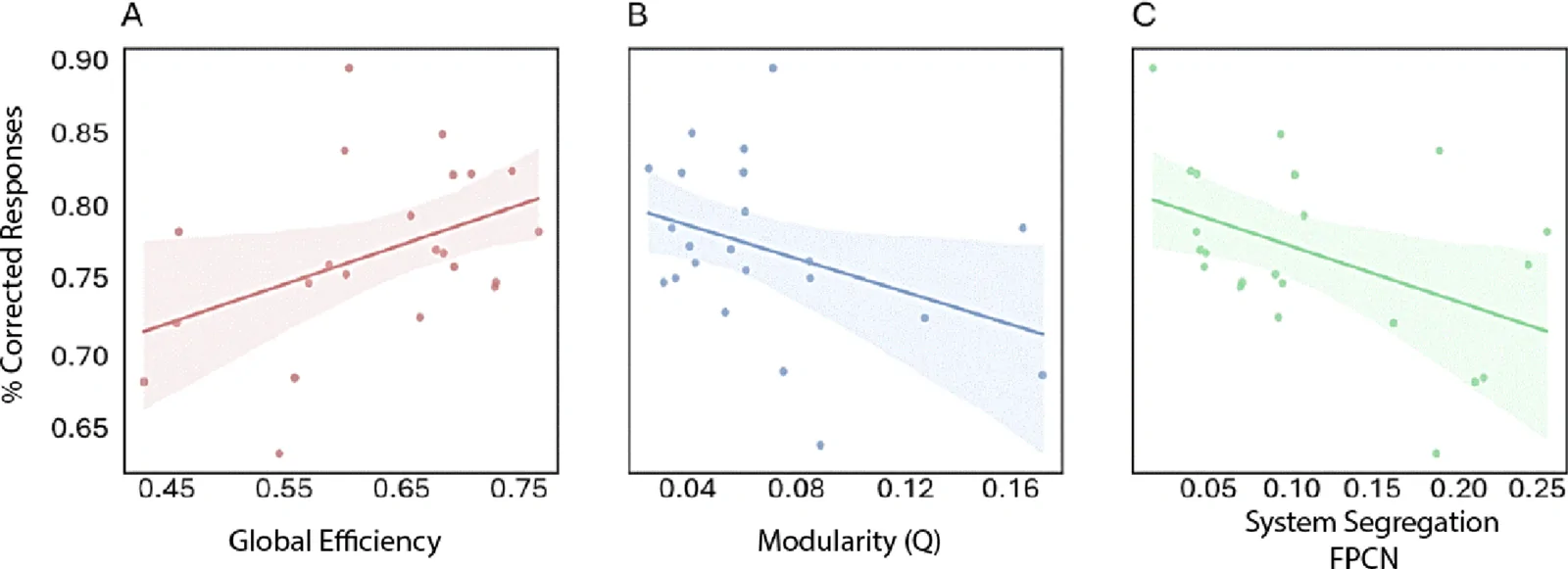
Scatterplots illustrating the association between memory accuracy (y-axis) and graph theory measures (x-axis): global efficiency (**A**), modularity (**B**), and system segregation of the Frontoparietal Control Network (**C**). Each dot is a subject; shaded areas represent 95% confidence intervals

Finally, at the node-wise level, no significant correlations were observed between performance and nodal measures of either the left or right hippocampus (all *p* > 0.05). In particular, path length was negatively correlated with accuracy for both the right (*r* = − 0.397, *p* = 0.067) and left hippocampus (*r* = − 0.334, *p* = 0.129). A similar result was found for degree centrality and nodal efficiency, with a positive non-significant correlation in both the right (degree centrality: *r* = 0.370, *p* = 0.090; nodal efficiency: *r* = 0.361, *p* = 0.099, respectively) and left hippocampus (degree centrality: *r* = 0.319, *p* = 0.148; nodal efficiency: *r* = 0.370, *p* = 0.090, respectively). Significant results are reported in Fig. 2.

### Network organization for different memory dimensions

**Fig. 3:**
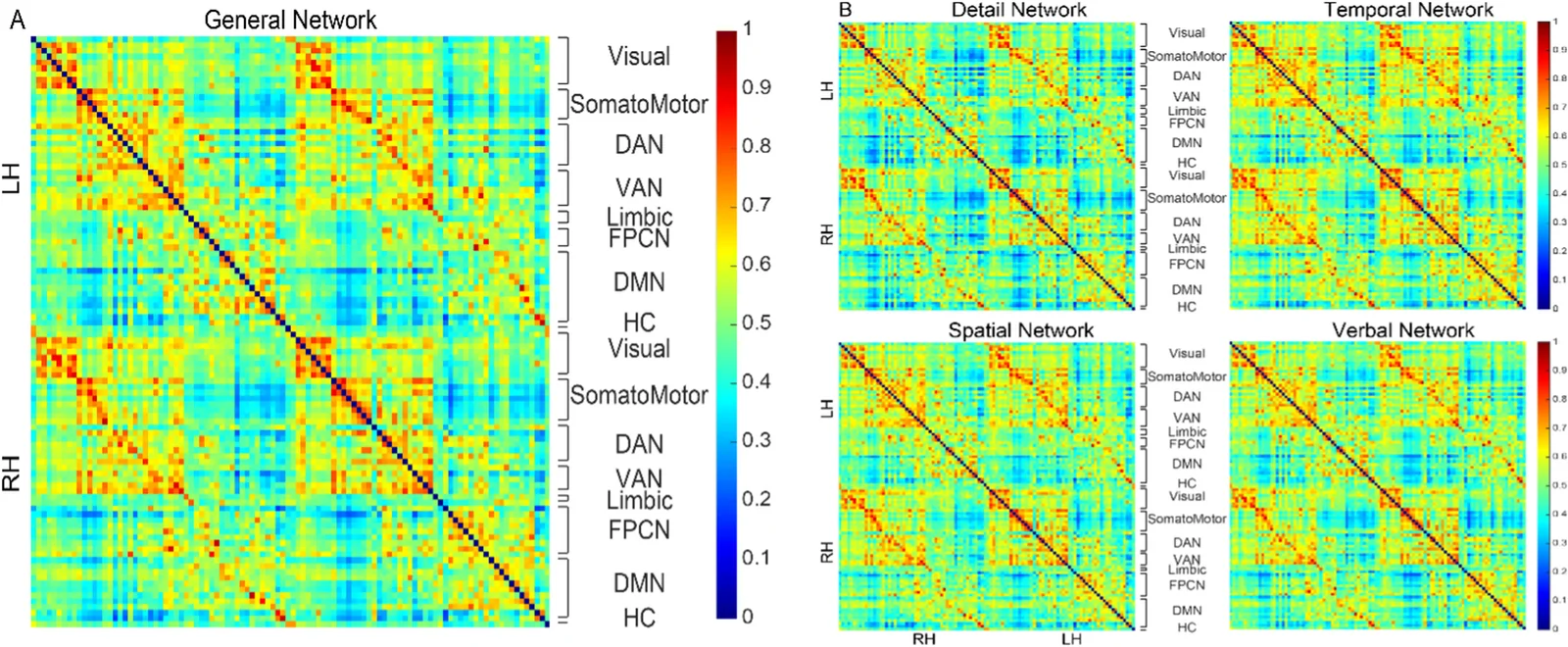
Average (across subjects) adjacency matrices derived from β series correlations are presented for all dimensions (panel **A**) and for each memory dimension and split by hemisphere (right and left)(panel **B**). For ease of visualization, regions of interest are ordered in accordance with the Schaefer atlas (Schaefer et al. 2018; Yeo et al. 2011) across networks (visual, somatomotor, dorsal-attention (DAN), visual-attention, limbic, Frontoparietal control (FPCN) and Default Mode network (DMN), with the left and right hippocampus defined using the Harvard - Oxford Cortical Structural Atlas (RRID: SCR_001476) (2025)

To assess the pattern of functional connectivity underlying cued recollection for different memory dimensions, we computed beta series correlation matrices across all correct trials and separately for each dimension (Fig. 3A, B).

The linear mixed-effects model showed no significant influence of Dimension on Global Efficiency (LR = 5.06, df = 3, *p* = 0.167), with global efficiency remaining comparable across conditions: details (M = 0.519, SD = 0.093), spatial (M = 0.543, SD = 0.109), temporal (M = 0.551, SD = 0.101), and verbal (M = 0.542, SD = 0.120), and behavioral accuracy did not predict global efficiency (b = 0.126, z = 1.03, *p* = 0.304). No significant effect between details and temporal condition was found (b = 0.040, z = 2.204, p_fdr = 0.1656). Similar results were obtained with a classical repeated-measures ANOVA with dimension as a factor (see Supplementary Materials).

A similar pattern emerged for Modularity. The linear mixed-effects model revealed no significant effect of Dimension (LR = 4.02, df = 3, *p* = 0.260), with stable values across conditions: details (M = 0.077, SD = 0.041), spatial (M = 0.067, SD = 0.045), temporal (M = 0.066, SD = 0.041), and verbal (M = 0.073, SD = 0.049), with behavioral accuracy did not predict modularity (b = − 0.102, z = − 0.77, *p* = 0.44). Again, no significant effect between the details and temporal condition was revealed (b = − 0.013, z = − 1.74, p_fdr = 0.389). To identify the best modular partition to serve as a template, the modularity algorithm was run 1,000 times on the average (across participants) network. Across dimensions, the three-module solution consistently revealed asymmetries in community size (see Supplementary Material). Similar results were obtained with the ANOVA approach (see Supplementary Materials).

**Fig. 4:**
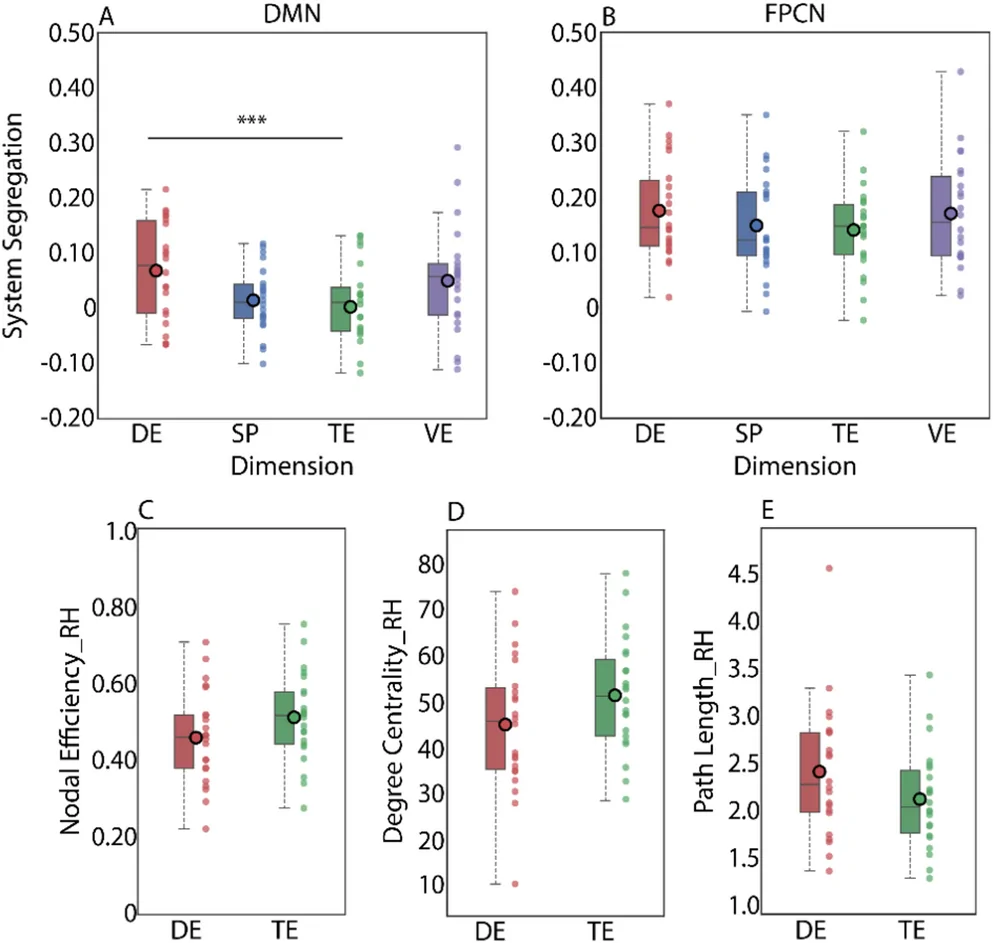
Boxplots illustrating system segregation for Default Mode Network (DMN) Frontoparietal control network (FPCN) (Panel **A**, **B**) for all dimensions. Panel **C**–**E**: Nodal Efficiency, Degree Centrality and Path Length for the Right Hippocampus in details (DE) and temporal (TE) dimensions. The median and the mean are represented by a horizontal line and a circle inside the box, respectively. The lower end of the box indicates the 1 st quartile while the upper end is the 3rd quartile

A different picture emerged at the network-community level. For the DMN, the linear mixed-effects model shown a significant effect of Dimension on DMN system segregation (LR = 10.03, df = 3, *p* = 0.018). The pairwise comparisons revealed a significantly greater segregation for the details condition compared to the temporal condition (b = 0.061, z = 2.67, p_fdr = 0.039), whereas no other pairwise comparisons reached significance (all p_fdr > 0.12). Importantly, behavioral accuracy did not significantly predict DMN segregation (b = 0.087, z = 0.66, *p* = 0.509). In contrast, no significant effect of Dimension was found for FPCN system segregation (LR = 5.17, df = 3, *p* = 0.160), with comparable values across conditions: details (M = 0.177), spatial (M = 0.150), temporal (M = 0.142), and verbal (M = 0.171) (Fig. 4A and B). The contrast between the details and temporal condition was not significant (b = − 0.041, z = − 2.01, p_fdr = 0.27). Similar results were obtained with the ANOVA approach (see Supplementary Materials).

Finally, no significant effect of Dimension was found for the left hippocampus across the three indices. Specifically, the linear mixed-effects model did not identify effects of Dimension for path length (LR = 4.58, df = 3, *p* = 0.206), degree centrality (LR = 4.87, df = 3, *p* = 0.181), or nodal efficiency (LR = 5.19, df = 3, *p* = 0.159). FDR-corrected pairwise comparisons between the details and temporal conditions indicated no significant difference in any of the three indices: path length (b = − 0.207, z = − 2.01, p_fdr = 0.266), degree centrality (b = − 4.859, z = − 2.06, p_fdr = 0.235), and nodal efficiency (b = − 0.047, z = − 2.17, p_fdr = 0.181). Similar results were obtained with the ANOVA approach (see supplementary Materials).

In contrast, for the right hippocampus, a significant effect of Dimension emerged for path length (LR = 10.15, df = 3, *p* = 0.017) and degree centrality (LR = 8.68, df = 3, *p* = 0.034). FDR-corrected pairwise comparisons revealed only a significant difference between the details and temporal conditions for the two indices: path length (b = 0.358, z = 3.10, p_fdr = 0.011), degree centrality (b = − 7.370, z = − 3.01, p_fdr = 0.016), whereas no other pairwise comparisons reached significance in both indices (all p_fdr > 0.05). A trend effect of Dimension was found for nodal efficiency (LR = 7.51, df = 3, *p* = 0.057), with planned comparison between details and temporal dimensions showing a significant difference (b = − 0.061, z = − 2.79, p_fdr = 0.031). The results indicated shorter path length, higher degree centrality, and higher nodal efficiency for the temporal compared to the details condition. Importantly, behavioral accuracy did not significantly predict any nodal index (all *p* > 0.05) (Fig. 4C-D-E). The ANOVA approach only indicated a trend towards a significant effect of length [F(3,63) = 2.55, n^2^ = 0.108, *p* = 0.064] and degree centrality [F(3,63) = 2.45, n^2^ = 0.104, *p* = 0.072], but not for nodal efficiency [F(3,63) = 2.11, n^2^ = 0.091, *p* = 0.108]. However, planned comparisons revealed a significant effect of condition for the three indices (see Supplementary Materials).

## Discussion

Previous research has linked successful retrieval to a functional brain network configuration characterized by a decrease in network modularity and an increase in communication efficiency, with a predominant role of the hippocampus. Here, we extended the framework to the cued recollection of complex audiovisual narratives, focusing on different memory dimensions and examining how topological shifts manifest across hierarchical scales, from global integration to network-specific segregation and hippocampal centrality. Firstly, we hypothesized that individuals who showed better overall cued recollection performance would be characterized by a more globally integrated brain architecture, mirroring patterns observed in more traditional retrieval paradigms. Secondly, we predicted that stronger network integration may be associated with the integration of information across multiple events, compared to judgments about single objects or characters, which rely on isolated, feature-specific details.

Consistent with our hypothesis, the inter-individual difference in cued recollection performance was positively associated with differences in global efficiency and modularity at the whole‑network level. Participants who showed better recollection performance were also characterized by lower segregation within the Frontoparietal control, but not the Default Mode network, suggesting that the FPCN loosens its boundaries to coordinate the activity of other networks (Spreng et al. 2010; Sestieri et al. 2014). Furthermore, the analysis revealed that retrieval conditions differing in relational demands (i.e. temporal dimensions) were characterized by lower system segregation in the DMN and enhanced nodal properties (higher degree centrality, higher efficiency, and shorter path length) of the right hippocampus. Overall, the present results suggest that successful recollection depends on system‑level integration, especially when reconstructing the complex relational structures required by temporal order judgments.

### Whole-brain integration and segregation in cued recollection

Our results indicate that better overall recollection performance is associated with a functional network architecture characterized by increased global integration and reduced modular organization. As a measure of the average inverse shortest path length, global efficiency provides a topological index of how swiftly and efficiently information is transmitted between distant brain areas (Bullmore and Sporns 2009). Greater efficiency likely facilitates parallel access to distributed representations, consistent with models of episodic reconstruction that emphasize the integration of perceptual, contextual, and self-referential details (Rugg and Vilberg 2013; Matyi and Spielberg 2022). For instance, Van den Heuvel et al. (2009) showed that individuals with more efficient resting-state networks perform better on tasks involving memory and executive control. Extending these findings, Geib et al. (2017a, b) reported that successful retrieval is associated with increased integration across the hippocampus, Default Mode Network, and Frontoparietal control systems. In addition, Shine and colleagues (2016) demonstrated that transient increases in global integration predict improved behavioral performance across cognitive tasks, underscoring the dynamic nature of network efficiency.

Unlike classic item recognition tasks, in the present paradigm the stimuli used to trigger memory retrieval (written sentences) did not directly reproduce the encoded audiovisual material. As noted by Mendelsohn et al. (2010), this type of retrieval, which was termed “cued recollection,” likely involves elaborative recall processes of imagery and scene reconstruction, rather than the selective reactivation of isolated memory components (Johnson et al. 1993; Rugg and Vilberg 2013). Another difference compared to more traditional paradigms, where successful retrieval (hits) and unsuccessful retrieval (misses) represent qualitatively distinct memorial outcomes, is that the present task requires participants to reconstruct complex scenes regardless of whether their eventual response is correct. As a consequence, scene reconstruction likely occurs also for incorrect responses, potentially reducing the difference in underlying network dynamics between the two trial types. Within this framework, our findings extend results from more traditional memory paradigms to a context that places greater demands on integrative processing. Indeed, despite the differences in task structure, the pattern we observe is broadly consistent with previous work showing that global network properties, such as global efficiency or modularity, relate to the quality of episodic retrieval (Richter et al. 2016; Shine et al. 2016).

Complementing these findings, we also observed a negative association between whole-brain modularity and overall recollection performance. Lower modularity reflects weaker boundaries between functional communities and enhanced intermodular communication, an architecture conducive to flexible coordination across systems (Shine et al. 2016). Indeed, while global efficiency reflects the capacity for large-scale integration, by capturing the efficiency of information transfer across distributed regions (Bullmore and Sporns 2009), modularity quantifies the degree of functional segregation by assessing the strength of community structure within the network (Newman 2006). Given that cognitive function depends on the balance between segregated and integrated processing (Friston 2009; Sporns 2016; Tononi et al. 1994), examining both metrics allows us to capture complementary aspects of task-related network organization. During retrieval, the brain may transiently shift toward an integration-dominant regime, enabling rapid interaction between cortical systems encoding perceptual and semantic features and hippocampal regions supporting pattern completion (Cooper and Ritchey 2019). Further evidence by Westphal et al. (2017) reported that participants with better recognition performance exhibited lower modularity and greater integration across key memory networks. Interestingly, this pattern contrasts with findings from resting-state studies, which have reported that higher modularity correlates with better performance on memory tasks administered outside the scanner (Stevens et al. 2012; Chan et al. 2014).

Beyond whole‑brain topology, our findings revealed a network‑specific dissociation that was reflected in task‑evoked network organization, with lower segregation within the FPCN associated with higher memory performance. This finding aligns with growing evidence that episodic retrieval depends on the flexible coupling between control and default networks, rather than their strict segregation (Spreng et al. 2010; Dixon et al. 2018; Westphal et al. 2017). In particular, if the FPCN becomes overly segregated, its functional specialization may come at the expense of cross-network communication, thereby reducing its ability to effectively coordinate with mnemonic and sensory systems. These results are consistent with the idea that the Frontoparietal Control Network supports cognitive control by dynamically reconfiguring its connectivity patterns, reducing internal segregation when cross-network coordination is required (Cole et al. 2013). Indeed, we found that this topological configuration tracked individual differences in retrieval performance (e.g., lower accuracy), underscoring its central role in supporting goal-directed mnemonic processing (Spreng et al. 2016; Westphal et al. 2017).

Another important aspect of the present findings concerns the role of the hippocampus within the functional architecture supporting cued recollection. As a matter of fact, at the node-wise level, no association emerged between individual differences in overall memory ability and measures of hippocampus centrality. This may appear in contrast with previous fMRI studies, which have consistently emphasized a key role of the hippocampus in episodic memory (Ranganath et al. 2004; Staresina et al. 2009; Rugg et al. 2012). One possible explanation for this negative result is the limited sample size of the current study (*N* = 22), which might have caused an underestimation of a potential relationship. However, we offer a different interpretation. While findings have been heterogeneous across studies, we note that univariate approaches alone cannot determine whether hippocampal engagement reflects sustained hub-like integration or more transient, content-dependent interactions within distributed networks. Indeed, contemporary models increasingly characterize the hippocampus as a transient conductor that initiates pattern completion and the reinstatement of event representations in the neocortex, and not as a stable integrative hub (Norman and O’Reilly, 2003; Rugg and Vilberg 2013; McCormick et al. 2018). This interpretation is consistent with graph-theoretical studies showing that recollection performance in similar paradigms is more strongly constrained by large-scale cortical integration than by nodal measures (Geib et al. 2017a, b; Westphal et al. 2017; Schedlbauer et al. 2014). From this perspective, our findings support the idea that, within cued recollection paradigms, the connectivity of the hippocampus is not directly related to retrieval performance. In other words, individual differences in recollection success appear more strongly constrained by large-scale cortical measures than by hippocampal nodal properties (Cooper and Ritchey 2019; Geib et al. 2017a, b; Westphal et al. 2017). This is consistent with models positioning the hippocampus as an initial modulator of retrieval, with a transient and content-dependent contribution (McCormick et al. 2018; Norman and O’Reilly, 2003; Rugg and Vilberg 2013).

### Network properties associated with different memory dimensions

Prior univariate fMRI works have shown that episodic retrieval flexibly adapts to task demands, with temporal judgments engaging broader network integration across DMN and hippocampal circuits (Kwok et al. 2012; Foudil et al. 2020; Liu et al. 2022; Jiang et al. 2025), whereas the retrieval of object or character details relies on more localized, modality-specific representations and increased segregation within posterior DMN regions (Westphal et al. 2017; Pan et al. 2025). This interpretation aligns with the idea that the DMN supports both integrative and specialized functions depending on task demands (Ranganath and Ritchey 2012). The greater segregation observed in the object/character details demands may therefore reflect a more encapsulated processing mode optimized for accessing fine-grained perceptual features, whereas retrieval conditions requiring the relational binding of multiple events may be associated with a more distributed, integrative configuration within the Default Mode network.

Temporal retrieval is not simply a more effortful version of the same process but may instead be associated with retrieval conditions that vary in their relational demands: judgments requiring the binding of multiple event representations into a relational sequence differ, at least in principle, from those involved in retrieving a single episodic feature (i.e. details) (Kwok and Macaluso 2015; Foudil et al. 2020; Procida et al. 2025). In addition, as highlighted through our univariate analyses included in our previous manuscript on this dataset (Procida et al. 2025), the temporal context engages partially distinct DMN subregions compared to other memory context, suggesting qualitatively different mnemonic processing rather than a generic increase in effort. This suggests that the shift toward a less segregated network state might not be a general consequence of the retrieval task but would reflect the computational requirements of the information being retrieved, with integrative dimensions potentially eliciting a more pronounced integration of the underlying functional architecture. Furthermore, the recollection of temporal information may engage a more distributed, integrative configuration to support the reconstruction of event sequences in the Default Mode Network, a system whose functional repertoire extends beyond memory retrieval to encompass mentalizing and Theory-of-Mind processes (Spreng and Grady 2010; Buckner et al. 2008; Spreng et al. 2009; Andrews-Hanna et al. 2010). It is therefore plausible that scenes involving social interactions may have elicited some degree of spontaneous mentalizing. In our paradigm, however, such contributions would be expected primarily for the verbal dimension, which include statements referring to characters’ speech or intentions. By contrast, the temporal-order and detail dimensions relied on descriptive, non-social statements requiring goal-directed evaluation of specific propositional content, a process known to attenuate spontaneous mentalizing (Christoff et al. 2009).

The fact that the temporal dimension was associated with lower accuracy raises the possibility that the observed network differences partly reflect retrieval difficulty or cognitive effort rather than dimension-specific mnemonic processes. However, the brain behavior correlations indicated that better performance across participants was associated with lower modularity and less segregation, i.e. in the opposite direction (Westphal et al. 2017). Moreover, in contrast with the results observed for the DMN, the absence of significant effects within the FPCN aligns with accounts proposing a general role for the FPCN during memory retrieval (Procida et al. 2025; Cabeza et al. 2008; Ritchey and Cooper 2020). This is consistent with the idea that the engagement of this network primarily reflects the way in which information is used to meet the retrieval task demands, rather than the retrieval process itself (Sestieri et al. 2017).

Whereas episodic memory is subserved by complex neural interactions and the continuous exchange of information between circuits distributed across several brain regions, the hippocampus stands out as the critical structure for the encoding, storage, and retrieval of such memories (Battaglia et al. 2011; Watrous et al. 2015). Several recent studies have extended task-based functional connectivity analyses by examining changes in bivariate connections (King et al. 2015) or basic graph-theoretical measures (Schedlbauer et al. 2014) between small subsets of memory-related regions. Of relevance to the current study, Schedlbauer et al. (2014) demonstrated that regions within the medial temporal lobe, including the hippocampus, exhibited greater connectivity and were more frequently positioned along shortest paths during successful retrieval, underscoring the importance of integrative processes. In this frame, our findings suggest that the right hippocampus displayed greater communication efficiency with the network (higher efficiency and shorter path length) and may have become a more convergent structure for information integration (higher centrality measures) under retrieval conditions with greater relational demands (i.e., temporal order judgments), consistent with the possibility that its functional integration scales with the amount of relational binding required by the retrieval demand. Recent evidence highlights the role of the right hippocampus in temporal memory. In particular, Liu et al. (2022) and Jiang et al. (2025) demonstrated that hippocampal activity, especially in the right hemisphere, tracks the structure and order of events during a memory task. Notably, this also aligns with a specific degree of lateralization to the type of information being remembered (Guerin and Miller 2009; Frisoni et al. 2025). While we maintain that the hippocampus is a critical structure for memory retrieval, we propose that its functionality alone cannot fully account for successful episodic recall; rather, its effectiveness depends on dynamic integration with broader cortical and subcortical networks, particularly when reconstructing the temporal structure of events.

### Study limitations and future directions

A few methodological considerations should be acknowledged to contextualize the present findings. A first limitation concerns the fact that the four memory dimensions were not purely mnemonic manipulations but were instantiated through sentences that necessarily differed in linguistic structure and in the type of relational processing they required (see Supplementary Material). As a result, the present design cannot disentangle the mnemonic dimension of interest from these intrinsic linguistic and relational-processing differences. We therefore cannot exclude the possibility that the network differences reported here partly reflect these linguistic and relational-processing differences, rather than the mnemonic dimension per se, and we caution against interpreting them as a pure index of dimension-specific mnemonic processing.

A second limitation concerns the restriction of the subcortical node set to the hippocampus. In the present study, we deliberately limited the subcortical component of the graph to the hippocampus since it plays a central role in episodic recollection and has been repeatedly identified as a hub whose dynamic integration with cortical networks predicts memory performance (Schedlbauer et al. 2014; Geib et al. 2017a, b). Including other subcortical nuclei, whose role on recollection is less clear, would have substantially increased the number of nodes in absence of a strong rationale. We nonetheless acknowledge that excluding the rest of the subcortex may influence graph-theoretic metrics such as modularity and integration, given that network topology is sensitive to the completeness of the node set. Future studies should consider extending the network model to include a broader set of subcortical regions, which would provide a more complete picture of the functional architecture underlying multidimensional recollection.

A third limitation concerns the absence of a direct comparison between encoding- and retrieval-related network organization. Although encoding-phase data were acquired, the present study focused exclusively on retrieval-related network topology, and we therefore cannot determine whether the individual differences observed during recollection reflect processes that emerge specifically at retrieval or instead originate from variability in how participants encoded the movie on the previous day. It remains possible that participants who formed stronger or more coherent memory representations during encoding also exhibited more integrated or more segregated network configurations during retrieval, creating an apparent brain–behavior relationship that partly reflects upstream variability in memory formation rather than retrieval mechanisms per se. A direct comparison between encoding and retrieval network configurations represents therefore a promising avenue for future analyses, which could clarify whether the dimension-specific modulation of DMN segregation and hippocampal topology observed here reflects a retrieval-specific reorganization or a pattern already established during the initial experience of the naturalistic material.

Furthermore, a methodological consideration concerns the use of a canonical hemodynamic response function (HRF) in the GLM used to estimate trial-wise beta values. Although more flexible approaches, such as finite impulse response (FIR) models, can better characterize task-evoked BOLD dynamics, the design of the present study-imposed constraints that made such approaches impractical. In particular, the retrieval window was fixed at 6 s to equate the temporal opportunity for memory search across dimensions and minimize the contribution of motor preparation, thereby eliminating the response-time variability required by FIR models for the reliable deconvolution of overlapping response. While we acknowledge that the use of a canonical HRF may obscure contributions from processes such as visual attention or linguistic analysis, we stress that our primary contrasts compared conditions matched for sensory input and response demands, differing principally in the mnemonic component of interest.

## Conclusions

The present study investigated how large-scale functional network organization supports cued recollection of naturalistic events across different memory dimensions. Greater memory performance was associated with higher global efficiency and lower modularity, as well as lower segregation within the Frontoparietal control network. Retrieval conditions placing greater relational demands (temporal order judgments), relative to those emphasizing single-item details, were characterized by lower segregation within the Default Mode network and increased degree centrality, efficiency, and reduced path length in the right hippocampus. These findings indicate that episodic retrieval relies on flexible network reconfiguration, and that retrieval conditions varying in mnemonic content and relational demands are associated with partially different topological patterns.

## Supplementary Information

Below is the link to the electronic supplementary material.

Supplementary Material 1

## Data Availability

The data that support the findings of this study are available on request from the corresponding author. The data are not publicly available due to privacy or ethical restrictions.
