# Supplementary material for: Multiscale network reorganization during the multidimensional recollection of naturalistic events
Source: Brain Struct Funct. 2026 Aug 27;231(7):126. doi: 10.1007/s00429-026-03184-8 (PMC13522023; doi:10.1007/s00429-026-03184-8)
Supplement: Supplementary file 1 [file 429_2026_3184_MOESM1_ESM.docx]

**Supplementary Materials**

Repeated measures ANOVAs across memory dimensions

To examine differences across memory dimensions for each graph-theoretical measure, repeated-measures ANOVAs with dimension as a factor were conducted for each index of interest. When the dimension effect was significant, post hoc analysis was performed. In case of non-significant effects, a planned paired-samples t-test directly contrasting two specific dimensions of interest (TE vs. DE dimensions) were conducted for each index, following the hypothesis stated in the introduction. Resulting p-values were corrected for multiple comparisons using FDR (α = .05, k = 6).

Repeated-measures ANOVAs revealed no significant effect of dimension on global efficiency [F (3,63) = 1.36, η² = 0.014, *p* = 0.265], with comparable values across conditions: details (M = 0.519, SD = 0.093), spatial (M = 0.543, SD = 0.109), temporal (M = 0.551, SD = 0.101), and verbal (M = 0.542, SD = 0.120). Planned sample t-test contrasting details and temporal condition did not reveal significant differences [t (21) = −2.03, p = 0.055, p_fdr = 0.330, Cohen’s d = −0.043] (Supplementary Figure 1A).

No significant effect was also found for modularity [F (3,63) = 1.29, η² = 0.012, *p* = 0.285], with stable values across dimensions: details (M = 0.077, SD = 0.041), spatial (M = 0.067, SD = 0.045), temporal (M = 0.066, SD = 0.041), and verbal (M = 0.073, SD = 0.049). No significant effect was observed between details and temporal conditions [t (21) = 1.82, p = 0.084, p_fdr = 0.252, Cohen’s d = 0.039] (Supplementary Figure 1B).

At the network community level, a significant effect of dimension emerged for DMN system segregation [F (3,63) = 3.714, η² = 0.150, p = 0.016], with post hoc tests showing greater segregation in the details than in the temporal condition [t (21) = 2.946, Cohen's d = 0.770, p = 0.008, p_fdr = 0.027], whereas no effect was found for FPCN system segregation [F (3,63) = 1.545, η² = 0.069, p = 0.212]. Paired t-test showed no differences between detail and temporal condition [t (21) = 1.86, p = 0.076, p_fdr= 0.244, Cohen’s d= 0.40]

Moreover, for the left hippocampus, no significant effect of dimension emerged for any index of interest: path length [F (3,63) = 1.67, η² = 0.071, p = 0.197], degree centrality [F (3,63) = 1.70, η² = 0.075, p = 0.176], and nodal efficiency [F (3,63) = 1.73, η² = 0.076, p = 0.170]. Paired t-tests comparing details and temporal dimension did not yield significant differences for any index [path length: [t (21) = 2.156, p = 0.043, p_fdr= 0.257]; degree centrality: [t (21) = -2.301, p= 0.032, p_fdr= 0.190; nodal efficiency: [t (21) = -2.338, p= 0.029, p_fdr= 0.176]. Contrary, ANOVAs indicated only indicated a trend towards a significant effect of path length [F (3,63) = 2.55, n^2^ = 0.108, p = 0.064] and degree centrality [F (3,63) = 2.45, n^2^ = 0.104, p = 0.072], but not for nodal efficiency [F (3,63) = 2.11, n^2^ = 0.091, p = 0.108]. for the right hippocampus. However, planned comparisons between the details and temporal conditions revealed significant differences for all three indices: path length [t (21) = 3.237, p = 0.004, p_fdr = 0.006, Cohen's d = 0.690], degree centrality [t (21) = −3.372, p = 0.003, p_fdr = 0.006, Cohen's d = −0.719] and nodal efficiency [t (21) = −3.087, p = 0.006, p_fdr = 0.006, Cohen's d = −0.658] (see Supplementary Figure 1C-D-E).

*Supplementary Figure 1. Boxplots illustrating Global Efficiency and Modularity (Q) for all memory dimensions (DE = details; SP = spatial; TE = temporal; VE = verbal) are shown in Panels A and B, respectively. In each boxplot, the median is indicated by a horizontal line, while the mean is represented by a circle. The lower and upper bounds of the box correspond to the first and third quartiles, respectively. Panels C–E show bar plots of Path Length, Degree Centrality, and Nodal Efficiency for the left and right hippocampus in the details (DE), spatial (SP), temporal (TE) and verbal (VE) dimensions. Bars represent mean values, and error bars indicate the standard error of the mean (SEM).*

Modularity Analysis: Community Partitioning Across Memory Dimensions

To identify the best modular partition to serve as a template, the modularity algorithm was run 1,000 times on the average network (across participants) for each memory dimension. Three different functional communities (i.e., modules) were identified from the modularity analysis. Because module labels are not directly comparable across dimensions, we report the relative size of each community for descriptive purposes. For the details dimension, community 1 included 35.29% of nodes, whereas community 2 and 3 included 38.24%, and 26.47%, respectively. The spatial dimension presented the three communities as organized: 31.37%, 41.18%, and 27.45% of nodes, respectively. The temporal dimension showed the lower percentage (18.63%) of nodes for the community 1, and 35.29%, and 46.08% for community 2 and 3. Finally, for the verbal dimension, the three communities included 31.37%, 42.16%, and 26.47% of nodes, respectively.

As shown in Figure 2, although a comparable number of communities were identified across dimensions, the composition of these modules varied, with several regions changing community organization between conditions. Such reorganization may reflect the flexible recruitment of large-scale functional systems to support distinct cognitive demands associated with each memory dimension. Notably, despite the variability in community composition across memory dimensions, the hippocampus was consistently assigned to the same community regardless of the memory dimension considered. This finding suggests that the hippocampus occupies a stable functional niche within the memory network, maintaining its community allegiance irrespective of the specific mnemonic demands imposed by each dimension.

*Supplementary Figure 2. Chord diagrams illustrate community structure of the functional connectivity network across four memory dimensions (Details, Spatial, Temporal, Verbal). Each segment on the outer ring represents a functional network, color-coded by network identity (see legend). The thin outer band indicates module membership (M1; M2; M3), as derived from group-level Louvain community detection. Chords connect networks belonging to the same module. Percentage labels indicate the proportion of nodes assigned to each module out of the total number of nodes (N= 102, 100 cortical parcels from the Schaefer Atlas and 2 bilateral hippocampal nodes from the Harvard Atlas). VAN = Visual Association Network; DAN = Dorsal Attention Network; Salience = Salience/Ventral Attention Network; SomMot = Somatomotor Network; Limbic = Limbic Network; FPCN = Frontoparietal Control Network; DMN = Default Mode Network; HC = Hippocampus*.

Linguistic analysis of the statements triggering cued recollection.

To examine whether sentences differed systematically in their linguistic properties across memory dimensions, we compared sentence length (number of words), part-of-speech composition (verb and noun counts), and word frequency across the four conditions. Word frequency was estimated using the ItWaC corpus (Baroni et al., 2006, 2009), a large-scale Italian web corpus (~1.9 billion tokens); each sentence was tokenized, words were matched to their corpus frequency, and sentence-level indices were computed (mean frequency, mean log-frequency, minimum frequency, and rare-word ratio). Average sentence length was 8.8 ± 1.3 words for object/character sentences, 9.2 ± 1.2 for spatial, 9.7 ± 1.4 for temporal, and 9.7 ± 1.3 for verbal sentences. A repeated measure ANOVA revealed a significant main effect of dimension (F (3,285) = 10.8, p < .001), with post-hoc tests indicating longer sentences for temporal and verbal dimensions compared to details and spatial (all p < .05; Supplementary Figure 3A). Verb count also differed significantly across dimensions (F (3,25) = 44.6, p < .001), with higher verb counts in temporal and verbal compared to details and spatial (all p < .001; Supplementary Figure 3B). The number of nouns was higher in verbal sentences compared to spatial and temporal dimensions (p < .001; Figure 3C). Word frequency also differed significantly across conditions (Kruskal-Wallis H = 139.0, p < .001), with temporal and verbal sentences showing higher frequency values than detail and spatial sentences (all pairwise comparisons significant after FDR correction, all p < .01).

*Supplementary Figure 3. Linguistic analysis of stimulus sentences across memory dimensions. Bar plots show mean values (± SEM) for word count (A), verb count (B), and noun count (C) across the four memory dimensions (DE = details, SP = spatial, TE = temporal, VE = verbal). Asterisks indicate significant differences relative to the other conditions (* p < .05; *** p < .001).*

This variability followed the experimental manipulation: to direct retrieval toward a specific mnemonic dimension, the sentences necessarily had to be structured in ways that selectively ensure the type of information participants were required to retrieve. Object/character sentences rely on specific nouns and perceptual adjectives that are relatively rare in everyday language, whereas temporal and verbal sentences are built around high-frequency action and communication verbs. To quantify this conceptualization, we examined whether the observed part-of-speech profiles matched the theoretical demands of each dimension. Using spaCy (it_core_news_sm) , a free open-source library for Natural Language Processing in Python for morphosyntactic annotation. We predicted that object/character sentences would be adjective-heavy (attributing static perceptual properties), spatial sentences would be noun-heavy (encoding relations between referents), temporal sentences would be adverb-heavy (expressing event sequences and connectives such as 'then', 'before', 'while'), and verbal sentences would be verb-heavy (encoding communicative acts). The observed profiles confirmed all four predictions: object/character sentences ranked highest in adjective count (M = 0.96), spatial in noun count (M = 3.67), temporal in adverb count (M = 0.84), and verbal in verb count (M = 2.49), with all differences being significant (Kruskal-Wallis, all p < .001).

Analysis of graph theory indices during correct versus incorrect recollection

The present paradigm differs from classical item recognition paradigms, in which hits and misses represent qualitatively distinct memorial outcomes, in that participants were required to reconstruct complex scenes irrespective of response accuracy. Consequently, we expected scene reconstruction to occur even in incorrect trials. To test this, statistical comparisons between correct and incorrect recollection were performed using paired samples t-tests on each graph-theoretical measure.

Nonetheless, we tested the presence of differences between correct versus incorrect responses in the same graph-theoretic measures considered in the main analysis. A paired-samples t-test comparing correct and incorrect trials revealed no significant difference in global efficiency (correct: M = 0.529, SD = 0.099; incorrect: M = 0.537, SD = 0.104; t(21) = −0.528, p = .603, Cohen's d = −0.112), indicating that successful recollection was not associated with a systematic change in global integration at the whole-brain level. Similarly, no significant difference emerged for modularity between correct and incorrect trials (correct: M = 0.065, SD = 0.039; incorrect: M = 0.065, SD = 0.041; t(21) = −0.034, p = .973, Cohen's d = −0.007). Moreover, at the network-community level of system segregation, we did not find any significant difference between the correct and incorrect conditions in any of Yeo's 7 Networks (Visual, Somatomotor, Dorsal Attention (DAN), Ventral Attention (VAN), Limbic, Frontoparietal Control (FPCN), and Default Mode Network (DMN) (all p > .05).

For the hippocampal nodal measures, paired t-tests revealed no significant difference between correct and incorrect trials for both hemispheres. For the left hippocampus, path length did not differ significantly (correct: M = 2.360, SD = 0.806; incorrect: M = 2.282, SD = 0.684; t(21) = 0.764, p = .453, Cohen's d = 0.163), nor did degree centrality (correct: M = 48.997, SD = 13.579; incorrect: M = 48.947, SD = 15.604; t(21) = 0.025, p = .980, Cohen's d = 0.005). For the right hippocampus, path length was also comparable across correct and incorrect trials (correct: M = 2.260, SD = 0.649; incorrect: M = 2.282, SD = 0.684; t(21) = 0.236, p = .816, Cohen's d = 0.050), as was degree centrality (correct: M = 47.676, SD = 15.231; incorrect: M = 48.099, SD = 14.848; t(21) = −0.215, p = .832, Cohen's d = −0.046).

**Title:** Multiscale Network Reorganization During the Multidimensional Recollection of Naturalistic Events

**Authors:** Federica Procida^1,2^, Maria Giulia Tullo^1,2^, Matteo Frisoni^3^, Annalisa Tosoni^2,4^, Mauro Gianni Perrucci^1,2,5^, Antonello Baldassarre^1,2^, Carlo Sestieri^1,2^.

**Affiliations:**

^1^Department of Neuroscience, Imaging and Clinical Sciences, University G. d'Annunzio of Chieti-Pescara, Via dei Vestini 31, 66100, Chieti, Italy.

^2^ITAB Institute for Advanced Biomedical Technologies, University G. d'Annunzio of Chieti-Pescara, Via dei Vestini 31, 66100, Chieti, Italy.

^3^Department of Psychology, ‘Sapienza’ University of Rome, Italy, Via dei Marsi, 78, 00185 Rome, Italy.

^4^Department of Psychology (DiPSI), University G. d'Annunzio of Chieti-Pescara, Via dei Vestini 31, 66100, Chieti, Italy.

^5^UdA-TechLab, Research Center, University “G. d’Annunzio” of Chieti-Pescara, Chieti,

Italy.

**Supplementary Material**

o/c = objects and character’s details

s= spatial layout

t= temporal sequence

v= verbal dialogue

**Practice**

**# Intro**

o/c

John Watson ha una tazza e una mela per colazione

John Watson has a cup and an apple for breakfast.

Watson nel letto ha una espressione molto tormentata

Watson in the bed has a very troubled expression.

John Watson indossa una canottiera di colore bianco

John Watson is wearing a white colored tank top.

Si vede Watson che tiene il viso nascosto nel cuscino

Watson can be seen keeping his face hidden in the pillow.

s

La pistola è collocata all'interno di un cassetto

The gun is placed inside a drawer.

La scrivania di Watson è collocata al centro della stanza

Watson's desk is placed in the center of the room.

Il bastone di John Watson è collocato sotto il letto

John Watson's cane is placed under the bed.

Il letto di John Watson è affiancato ad una parete

John Watson's bed is flanked by a wall.

t

Prima si vede Watson seduto e poi sdraiato nel letto

First Watson is seen sitting and then lying in bed.

Durante le immagini di guerra si sentono urla e spari

Screams and gunfire can be heard during war images.

Si vedono scene di guerra poi Watson nel letto

War scenes are seen then Watson in the bed.

Watson guarda la pistola poi si vedono le scene di guerra

Watson looks at the gun then war scenes are seen.

**Retrieval**

**#1 Terapia (Therapy)**

o/c

La psicologa è una donna nera che porta i capelli corti

The psychologist is a black woman who wears her hair short.

La psicologa ha una espressione rilassata e comprensiva

The psychologist has a relaxed and understanding expression.

La psicologa indossa un paio di occhiali di colore arancione

The psychologist wears a pair of orange-colored glasses.

La psicologa è una donna anziana sulla sessantina

The psychologist is an elderly woman in her sixties.

s

Watson e la psicologa sono seduti al centro della stanza

Watson and the psychologist are sitting in the center of the room.

Watson e la psicologa sono seduti uno di fronte all'altra

Watson and the psychologist are sitting across from each other.

Watson siede dando le spalle alla finestra principale

Watson sits with his back to the main window.

La psicologa è seduta dietro la sua scrivania

The psychologist is sitting behind her desk

t

Il dottor Watson si schiarisce la voce mentre sta parlando

Doctor Watson clears his throat as he is speaking.

Watson e la psicologa si interrompono a vicenda parlandosi sopra

Watson and the psychologist interrupt each other by talking over.

Watson e la psicologa sono di profilo poi c'è uno zoom su di lui

Watson and the psychologist are in profile, then there is a zoom in on him.

Dopo il dialogo la scena finisce con un lungo silenzio da parte di entrambi

After the dialogue the scene ends with a long silence from both of them.

v

La psicologa dice che Watson controlla quello che lei scrive

The psychologist says Watson controls what she writes.

La psicologa dice che Watson deve riadattarsi alla vita civile

The psychologist says Watson needs to readjust to civilian life.

Watson dice che la scrittura del blog sta andando male

Watson says blog writing is going poorly.

La psicologa dice che alla fine il blog non sarà utile

The psychologist says that ultimately the blog will be of no use.

**#2 Conferenza stampa della vedova (Widow's press conference)**

o/c

La vedova Patterson è una donna castana di mezza età

The widow Patterson is a middle-aged woman with brown hair.

La vedova Patterson legge il discorso con tono affranto

The widow Patterson reads the speech in a heartbroken tone.

L'amante viene visualizzata sullo schermo di un cellulare

The lover is displayed on a cell phone screen.

La vedova è una donna sciatta e robusta

The widow is a scruffy and rather robust woman

s

I giornalisti sono posizionati in gruppo dietro la vedova

The reporters are positioned in a group behind the widow's.

La foto del defunto è posizionata alle spalle della vedova

The photograph of the deceased is placed behind the widow's back.

L'amante è posizionata accanto alla foto del defunto

The lover is positioned next to the photo of the deceased.

La vedova del signore Jeffrey Patterson siede al tavolo

Sir Jeffrey Patterson's widow sits at the table

t

La vedova comincia a parlare soltanto alla fine della scena

The widow only begins to speak at the end of the scene.

L'amante compare prima della vedova che tiene il discorso

The lover appears before the widow who gives the speech.

Mentre la vedova parla i giornalisti ascoltano e scattano foto

As the widow speaks, reporters listen and take photos.

L’amante è in lacrime mentre la vedova parla

The lover is seen in tears while the widow is speaking.

v

La vedova dice che l'uomo è stato senza dubbio assassinato

The widow says the man was undoubtedly murdered.

La vedova dice che quella morte rimarrà un trauma per tutti

The widow says that death will remain a trauma for everyone.

La vedova Patterson dichiara che suo marito amava la vita

Widow Patterson declares that her husband loved life.

La vedova dice che l’uomo ha seri problemi a lavoro

The widow says the man had serious problems at work.

**#3 Sotto la pioggia (Under the rain)**

o/c

La vittima indossa un giubbotto senza cappuccio

The victim wears a hoodless jacket.

La vittima indossa un impermeabile lungo e chiaro

The victim wears a long, light-colored raincoat.

L'ombrello dell'amico è un modello piccolo

The friend’s umbrella is a small model.

L’amico della vittima ha i capelli totalmente rasati

The victim's friend has completely shaved hair.

s

Gli alberi del marciapiede sono tutti attaccati al muro

The trees on the sidewalk are all attached to the wall.

I due ragazzi sono ripresi dall'alto verso il basso

The two boys are filmed from top to bottom.

I ragazzi sono posizionati sul marciapiede sinistro

The boys are positioned on the left sidewalk.

L’arrivo del taxi è ripreso frontalmente

The arriving taxi is shot head-on.

t

I ragazzi camminano insieme prima che la vittima si allontani

The boys walk together before the victim walks away.

La vittima tenta di fermare un taxi poi va verso casa

The victim attempts to stop a cab and then heads home.

Uno prova a fermare un taxi mentre l’altro apre l’ombrello

One tries to stop the taxi then the other opens the umbrella

L'amico della vittima apre l'ombrello alla fine della scena

The victim’s friend opens the umbrella at the end of the scene.

v

La vittima dice che così finiranno per ammalarsi

The victim says they will end up getting sick this way.

La vittima saluta dicendo che si rivedranno al mattino

The victim greets saying they will see each other again in the morning.

L'amico propone di usare insieme un ombrello

The friend proposes to use an umbrella together.

La vittima dice si allontenerà per solo due minuti

The victim says he will only leave for two minutes.

**#4 Party (=)**

o/c

La vittima appare come una donna bionda di mezza età

The victim appears as a middle-aged blond woman.

La vittima è vestita in modo trasandato e con vestiti strappati

The victim is dressed shabbily and in torn clothes.

L'uomo indossa un completo in giacca e cravatta

The man is wearing a suit and tie.

L’uomo indossa un lungo impermeabile viola

The man is wearing a long purple raincoat.

s

La vittima in lacrime si trova presso una zona con dei containers

The tearful victim stands at an area with containers.

Le chiavi della vittima sono appoggiate sopra un tavolino

The victim's keys are resting on top of a small table.

L'uomo e la donna dialogano in prossimità del bancone

Man and woman dialogue near the counter.

La vittima in lacrime si trova presso una zona con dei containers

The crying victim is found near an area with containers

t

Durante tutto il dialogo tra i due si sente la vittima piangere

Throughout the dialogue between the two the victim is heard crying.

La donna arriva al bancone e ha un dialogo con l'uomo

The woman arrives at the counter and has a dialogue with the man.

Prima c'è il dialogo tra l'uomo e la donna poi si vede la vittima

The victim is seen after the dialogue between the man and the woman.

Il dialogo tra l’uomo e la donna è alla fine della scena

The dialogue between the man and the woman is at the end of the scene

v

La donna dice che ha preso le chiavi della vittima dalla borsa

The woman says she took the victim’s keys from her purse.

La vittima in lacrime chiede disperatamente di avere pietà

The crying victim pleads for mercy.

L'uomo chiede informazioni alla donna circa la situazione

The man asks the woman about the situation.

L’uomo chiede dove è esattamente il killer

The man asks where exactly the killer is.

**#5 Conferenza stampa della polizia (Police press conference)**

o/c

I giornalisti nel pubblico sono vestiti in modo piuttosto elegante

The journalists in the audience are dressed rather elegantly.

Il sergente Donovan legge utilizzando degli occhiali da vista rossi

Sergeant Donovan reads using red eyeglasses.

L'ispettore Lestrade e il sergente Donovan indossano l'uniforme

Inspector Lestrade and Sergeant Donovan wear their uniforms.

Il telefono dell’inspettore Lestrarde è di colore scuro

Inspector Lestrade’s phone is dark-colored.

s

I giornalisti sono accalcati ai piedi del tavolo della conferenza

Journalists are crowded at the foot of the conference table.

Le foto delle tre vittime sono collocate alle spalle di Lestrade

Photos of the three victims are placed behind Lestrade's back.

L'ispettore Lestrade è posizionato di profilo rispetto ai giornalisti

Inspector Lestrade is positioned in profile in relation to reporters.

Le foto delle tre vittime sono alle spalle di Lestrade

The photos of the three victims are placed behind Lestrade.

t

I giornalisti leggono gli sms prima che inizi la conferenza stampa

The journalists read messages before the press conference begins.

Lestrade lascia la conferenza poi legge un messaggio di Sherlock

Lestrade leaves the conference then reads a message from Sherlock.

L'ispettore Lestrade e il sergente Donovan parlano a turno

Inspector Lestrade and Sergeant Donovan take turns speaking.

Prima parla il sergente Donovan e successivamente i giornalisti

Sergeant Donovan speaks first and then the journalists

v

Donovan dice che un cadavere è stato trovato fuori Londra

Donovan says that a dead body was found outside London.

Lestrade dichiara che la situazione non è seguita dagli investigatori

Lestrade states that the situation is not being followed by investigators.

Un giornalista commenta dicendo che non esistono suicidi in serie

A journalist comments saying that there are no serial suicides.

Il sergente Dovan richiede ai giornalisti di ignorare I messaggi

Sergeant Donovan requests reporters to ignore the messages.

**#6 Watson al parco (Watson at the park)**

o/c

La rivista di Mike Stamford sembra un giornale quotidiano

Mike Stamford’s magazine looks like a daily newspaper.

Mike Stamford è un uomo dall'aspetto sportivo

Mike Stamford is a sporty-looking man.

Mike Stamford ha uno zainetto rosso monospalla

Mike Stamford has a red one-shoulder backpack.

Mike Stamford è vestito in modo piuttosto elegante

Mike Stamford is dressed quite sharply

s

La panchina si trova nel parco affacciata al vialetto

The bench is located in the park facing the driveway.

Mike Stamford è ripreso dall'alto verso il basso

Mike Stamford is filmed from top to bottom.

Watson e Mike Stamford sono entrambi ripresi di spalle

Watson and Mike Stamford are both filmed from behind.

Watson e Mike Stamford sono seduti uno accanto all’altro

Watson and Mike Stamford are sitting next to each other

t

Mike Stamford beve una bibita e poi incontra Watson

Mike Stamford drinks a soda and then meets Watson.

Watson cammina col bastone poi siede con Mike Stamford

Watson walks with a cane and then sits with Mike Stamford.

Watson stringe la mano a Stamford solo alla fine della scena

Watson shakes Stamford’s hand only at the end of the scene.

v

Stamford aveva saputo che Watson si era fatto sparare

Stamford had learned that Watson had gotten himself shot.

Stamford propone a Watson di prendersi un coinquilino

Stamford proposes that Watson get a roommate.

Watson si descrive come un coinquilino rumoroso

Watson describes himself as a noisy roommate.

Stamford non conosce nessuno in cerca di coinquilini

Stamford doesn't know anyone looking for roommates

**#7 Esperimenti all’obitorio (Experiments at the morgue)**

o/c

Il frustino di Sherlock è composto da numerosi lacci

Sherlock’s riding crop consists of numerous laces.

La busta contenente il cadavere è di colore nero

The bag containing the corpse is black.

L'assistente Molly ha i capelli castani legati in una coda

Assistant Molly has brown hair tied in a ponytail.

Sherlock sta indossando un lungo camice

Sherlock is wearing a long white coat

s

I tavoli dell'obitorio sono disposti lungo le pareti

Morgue tables are arranged along the walls.

Il cadavere è posizionato sopra un tavolo dell'obitorio

The corpse is placed on top a table in the morgue.

Il cadavere nell'obitorio viene ripreso dall'alto in basso

The corpse in the morgue is filmed from top to bottom.

L'assistente Molly si trova dall'altra parte della vetrata

Assistant Molly is on the other side of the glass window

t

Le frustate di Sherlock sono via via più lente e costanti

Sherlock’s lashing are gradually slower and more constant.

Sherlock frusta il cadavere poi Molly si presenta con il rossetto

Sherlock whips the corpse then Molly shows up with lipstick.

Sherlock Holmes frusta il cadavere e poi lo annusa

Sherlock Holmes whips the corpse and then smells it.

Sherlock apre la busta con il corpo dentro e lo odora

Sherlock opens the bag with the body and then smells it.

v

Molly dice che il corpo è stato recuperato in un incendio

Molly says the body was recovered in a fire.

Molly dice che il morto era una brava persona

Molly says the dead man was a good person.

Sherlock dice a Molly che prima non aveva il rossetto

Sherlock tells Molly that she did not have lipstick before.

Molly dice che il corpo è stato recuperato in un incendio

Molly says the body was recovered in a fire.

**#8 Conversazione in laboratorio (Conversation in the lab)**

o/c

Il cellulare di John Watson ha la tastiera orizzontale

John Watson’s cell phone has a horizontal keyboard.

L'assistente Molly porta il caffè in un thermos fumante

Assistant Molly brings coffee in a smoking thermos.

Sherlock Holmes indossa una giacca scura e la camicia

Sherlock Holmes wears a dark jacket and a shirt.

L'assistente Molly indossa una cuffia da laboratorio

Assistant Molly is wearing a lab cap

s

Il microscopio del laboratorio è sul tavolo centrale

The laboratory microscope is on the central table.

Watson è posizionato vicino alla porta di ingresso

Watson is positioned near the entrance door.

Watson e Stamford passeggiano intorno al tavolo da lavoro

Watson and Stamford walk around the work table.

Watson e Stamford passeggiano intorno al tavolo da lavoro

Watson and Stamford stroll around the worktable

t

Watson e Stamford entrano nella stanza poi arriva Sherlock

Watson and Stamford enter the room then Sherlock arrives.

Watson dà il telefono a Sherlock prima delle presentazioni

Watson gives the phone to Sherlock before introductions.

Watson parla con Sherlock poi arriva Molly con il caffè

Watson talks to Sherlock then Molly arrives with coffee.

Sherlock esce dalla stanza prima dell'assistente Molly

Sherlock leaves the room before assistant Molly

v

Sherlock chiede a Mike Stamford di prestargli il telefono

Sherlock asks Mike Stamford to lend him the phone.

Sherlock chiede se a Watson piace la chitarra heavy metal

Sherlock asks if Watson likes heavy metal guitar.

Sherlock chiede se Watson è stato in missione in Kosovo

Sherlock asks if Watson has been on a mission in Kosovo.

Sherlock dice che Molly ha la bocca troppo piccola

Sherlock says Molly's mouth is too small

**#9 Visita all'appartamento (Visit to the apartment)**

o/c

Il teschio dell'amico di Sherlock è completamente bianco

Sherlock’s friend's skull is completely white.

L'ispettore Lestrade indossa il cappello della polizia

Inspector Lestrade wears the police hat.

L'ispettore Lestrade indossa un cappotto di colore scuro

Inspector Lestrade wears a dark-colored coat.

La signora Hudson indossa una vestaglia di colore bianco

Mrs. Hudson is wearing a white robe

s

Il teschio è collocato per terra al centro della stanza

The skull is placed on the floor in the center of the room.

La signora Hudson parla con Watson dalla cucina

Mrs. Hudson talks to Watson from the kitchen.

Watson è dal lato opposto della stanza rispetto al camino

Watson is on the opposite side of the room from the fireplace.

Sherlock Holmes è posizionato in prossimità della finestra

Sherlock Holmes is positioned near the window

t

Sherlock Holmes sta saltando di gioia mentre entra Lestrade

Sherlock Holmes is jumping for joy as Lestrade walks in.

Sherlock vede la macchina della polizia poi arriva Lestrade

Sherlock sees the police car then Lestrade arrives.

Watson ha un dialogo da solo con la signora poi arriva Lestrade

Watson has a dialogue alone with the lady then Lestrade arrives.

Sherlock ritorna a sorpresa nella stanza mentre Watson legge

Sherlock unexpectedly returns to the room while Watson reads

v

Lestrade dice questa volta c'è un messaggio sul luogo del crimine

Lestrade says this time there is a message at the crime scene.

Sherlock Holmes dice che lui deve un favore alla signora Hudson

Sherlock Holmes says he owes Mrs. Hudson a favor.

Watson dice di non volere vedere mai più delle malattie

Watson says he never wants to see diseases again.

Watson racconta a Sherlock di averlo cercato su internet

Watson tells Sherlock that he looked for him on the internet

**#10 Sherlock e Watson in taxi (Sherlock and Watson in the cab)**

o/c

I graffi sul cellulare formano quasi un cerchio

The scratches on the cell phone almost form a circle.

I finestrini del taxi di Sherlock Holmes sono trasparenti

Sherlock Holmes' cab windows are transparent.

Il taxi con i due investigatori è fermo a motore spento

The taxi with the two detectives is stopped with the engine off.

Il cellulare con I graffi appare di colore scuro

The cell phone with scratches appears dark colored.

s

Il cellulare di Watson è posizionato sul sedile

Watson's cell phone is placed on the seat.

Il dottor Watson è seduto alla destra di Sherlock

Doctor Watson is sittting on the right of Sherlock.

Sherlock Holmes e Watson nel taxi sono ripresi di spalle

Sherlock Holmes and Watson in the cab are filmed from behind.

Sherlock e Watson sono entrambi seduti nei posti dietro

Sherlock and Watson are both sitting in the back seats.

t

I vari flashbacks si vedono soltanto alla fine della scena

The various flashbacks are only seen at the end of the scene.

Prima si vede il dottor John Watson nel taxi poi Sherlock

Dr. John Watson is shown first in the taxi, then Sherlock.

Watson dà il cellulare a Sherlock poi si vedono i suoi polsi

Watson gives cell phone to Sherlock then his wrists are seen.

Sherlock e Watson sono sul taxi poi si rivede il loro incontro

Sherlock and Watson are in the cab then we review their meeting

v

Sherlock dice che la polizia consulta anche i dilettanti

Sherlock says police consult amateurs as well.

Sherlock dice di essersi inventato quel tipo di lavoro

Sherlock says he invented that kind of work.

Sherlock è abituato a sentirsi dire che è straordinario

Sherlock is used to being told he is extraordinary.

Sherlock dice che la polizia spesso brancola nel buio

Sherlock says police often grope in the dark

**#11 Arrivo sulla scena del crimine (Arrival at the crime scene)**

o/c

Anderson indossa la tuta celeste per lo svolgimento delle indagini

Anderson wears the light blue suit when conducting the investigation.

Anderson indossa una mascherina per svolgere le indagini

Anderson wears a mask to carry out the investigation.

Il sergente Sally Donovan porta i capelli neri sciolti

Sergeant Sally Donovan wears her black hair down.

Il sergente Donovan porta degli occhiali speciali per le indagini

Sergeant Donovan wears special glasses for investigations

s

Anderson e il sergente Donovan sono una di fianco all'altro

Anderson and Sergeant Donovan stand side by side.

Anderson parla con Sherlock Holmes faccia a faccia

Anderson talks with Sherlock Holmes face to face.

Anderson, Watson e Sherlock sono sulla strada davanti all'edificio

Anderson, Watson, and Sherlock are on the road in front of the building.

Il sergente Donovan è posizionato all'interno dell'auto della polizia

Sergeant Donovan is inside the police car.

t

Il dottor John Watson conosce prima Donovan poi Anderson

Dr. John Watson first knows Donovan then Anderson.

Sherlock arriva di fronte all'ingresso poi parlano con Anderson

Sherlock arrives across the hall then talks to Anderson.

Sherlock entra nell'edificio poi parla con il sergente Donovan

Sherlock enters the building and then talks to Sergeant Donovan.

Prima Sherlock Holmes entra nell'edificio poi Anderson esce

First Sherlock Holmes goes into the building then Anderson exits

v

Anderson dice che Sherlock arriva sempre con nuovi colleghi

Anderson says Sherlock always arrives with new colleagues.

Sherlock chiede ad Anderson se sua moglie è via da molto

Sherlock asks Anderson if his wife has been gone long.

Sherlock dice che Anderson ha il profumo per donna di Donovan

Sherlock says Anderson has Donovan’s women's perfume.

Sherlock chiede se Donovan ha lucidato il pavimento di Anderson

Sherlock asks if Donovan polished Anderson's floor

**#12 Preparativi al piano terra (Preparations on the ground-floor)**

o/c

Le tute di Watson e Lestrade sono di colore differente

Watson and Lestrade’s suits are different colors.

Lestrade si protegge bocca e naso con una mascherina per indagini

Lestrade protects his mouth and nose with an investigation mask.

Sherlock Holmes indossa dei guanti attillati per le indagini

Sherlock Holmes wears tight-fitting gloves for investigation.

L'ispettore Lestrade indossa una tuta celeste completa

Inspector Lestrade wears a light blue suit.

s

I tre personaggi sono ripresi di spalle mentre indossano le tute

The three characters are filmed from behind while wearing suits.

Si vede il tavolo che è posizionato al centro di una stanza

The table is seen positioned in the center of a room.

Sulle scale Sherlock e Watson sono dietro all'ispettore Lestrade

On the stairs Sherlock and Watson are behind Inspector Lestrade.

Si vede il vestito del dottor John Watson posato sul tavolo.

Dr. John Watson's suit is seen being laid on the table.

t

L'ispettore Lestrade sale le scale poi Watson indossa la tuta

Inspector Lestrade climbs the stairs and then Watson puts on the suit.

Sherlock e Watson arrivano mentre Lestrade sta indossando la tuta

Sherlock and Watson arrive while Lestrade is wearing the suit.

Sherlock e Watson salgono le scale poi incontrano Lestrade

Sherlock and Watson climb the stairs and then meet Lestrade.

Lestrade parla con Sherlock mentre finisce di indossare la tuta

Lestrade talks to Sherlock as he finishes putting on his suit.

v

Lestrade dice che il corpo è lì da poco ed è stato trovato da ragazzini

Lestrade says the body has not been there for long and was found by kids.

Sherlock fa lo scambio di presentazioni tra Watson e Lestrade

Sherlock makes the exchange of introductions between Watson and Lestrade.

Sherlock Holmes dice all'ispettore Lestrade che Watson è con lui

Sherlock Holmes tells Inspector Lestrade that Watson is with him.

Lestrade dice a Sherlock che gli concede tutto il tempo che vuole

Lestrade tells Sherlock that he will give him all the time he wants.

**#13 Esame del cadavere (Examination of the corpse)**

o/c

Il cadavere indossa dei grandi orecchini d'argento

The corpse is wearing large silver earrings.

Le macchie dietro al polpaccio hanno la forma di piccoli punti

The spots behind the calf are in the shape of small dots.

Sherlock indossa la tuta protettiva per le indagini

Sherlock wears protective suit for the investigation.

Alcuni investigatori indossano il cappello da poliziotto

Some detectives wear police hats

s

Il volto del cadavere è girato in direzione della porta

The face of the corpse is turned in the direction of the door.

Il cadavere della donna è appoggiato ad una parete

The woman’s body is leaning against a wall.

L'ispettore Lestrade è dalla parte dei piedi del cadavere

Inspector Lestrade is on the side of the corpse’s feet.

Lestrade e Watson sono vicini alla ringhiera delle scale

Lestrade and Watson are near the stair railing.

t

Sherlock esamina la scritta sul pavimento poi l'anello

Sherlock examines the writings on the floor then the ring.

Sherlock manda via Anderson poi Watson esamina il corpo

Sherlock sends Anderson away then Watson examines the body.

Watson esamina il cadavere poi è il turno di Sherlock

Watson examines the corpse then it’s Sherlock’s turn.

Sherlock salta di gioia poi Lestrade esce dalla stanza

Sherlock jumps for joy then Lestrade leaves the room

v

Sherlock Holmes dice che i serial killers sono noiosi

Sherlock Holmes says serial killers are boring.

Sherlock ipotizza che la vittima lavorasse per la televisione

Sherlock speculates that the victim worked for television.

Watson ipotizza che la vittima sia morta per asfissia

Watson supposes that the victim has died from asphyxiation.

Sherlock chiede a Lestrade di dirgli tutto ciò che sta pensando

Sherlock asks Lestrade to tell him everything he's thinking

**#14 Watson e Donovan (Watson and Donovan)**

o/c

Il sergente Donovan indossa dei guanti protettivi attillati

Sergeant Donovan wears tight-fitting protective gloves.

Il sergente Donovan indossa delle scarpe con i tacchi

Sergeant Donovan wears shoes with heels.

Le automobili della polizia hanno i lampeggianti spenti

The police cars’ flashing lights are turned off.

Il sergent Donovan indossa una gonna nera

Sergeant Donovan wears a black skirt

s

Il sergente Donovan si trova vicino all'automobile della polizia

Sergeant Donovan stands near the police car.

John Watson e il sergente Donovan sono sul marciapiede

John Watson and Sergeant Donovan are on the sidewalk.

L'auto della polizia è posizionata non lontana dall'ingresso

The police car is positioned not far from the entrance.

Watson e Donovan hanno una conversazione in macchina

Watson and Donovan have a conversation inside the car.

t

Si sentono squilli poi Watson smette di parlare con Donovan

Rings are heard then Watson stops talking to Donovan.

Watson incontra il sergente Donovan poi esce dall'edificio

Watson meets Sergeant Donovan then exits the building.

Watson incontra Donovan mentre lei sta parlando al poliziotto

Watson meets Donovan while she talks to a policeman.

Watson parla con Donovan poi abbandona l'area delimitata

Watson talks with Donovan then leaves the demarcated zone.

v

Donovan dice di conoscere un crimine commesso da Sherlock

Donovan says she knows of a crime committed by Sherlock.

Donovan sostiene che Sherlock Holmes abbia numerosi amici

Donovan claims that Sherlock Holmes has numerous friends.

Donovan dice di conoscere un crimine commesso da Sherlock

Donovan says he knows of a crime Sherlock committed.

Il sergente Donovan sostiene che gli psicopatici si annoiano

Sergeant Donovan claims that psychopaths get bored.

**#15 Telefonata per Watson (Phone call for Watson)**

o/c

Gli squilli dei telefoni emettono un suono molto simile

Phone rings make a very similar sound.

John Watson indossa gli occhiali per vedere le telecamere

John Watson wears glasses to see the cameras.

Le videocamere mobili di Mycroft sono di colore nero

Mycroft’s mobile cameras are black in color.

La cabina rossa è composta anche da vetri trasparenti

The red cabin also consists of transparent glass.

s

Dentro la cabina il dottor Watson è rivolto verso la strada

Inside the cabin, Dr. Watson is facing the street.

La cabina telefonica rossa è alla destra del dottor Watson

The red phone box is to the right of Dr. Watson.

L'automobile con Anthea è alle spalle del dottor Watson

The car with Anthea is behind Doctor Watson.

Le telecamere di Mycroft riprendono John Watson dal basso

Mycroft’s cameras film John Watson from below.

t

John Watson chiama il taxi dopo avere parlato al telefono

John Watson calls a cab after talking on the phone.

John Watson entra nella cabina rossa poi squilla il telefono

John Watson enters the red booth then the phone rings.

Watson risponde al telefono poi si vedono le telecamere

Watson answers the phone then the cameras are seen.

Si vedono le telecamere e più tardi arriva l'auto con Anthea

Cameras are seen and later the car arrives with Anthea.

v

Mycroft è sicuro che Watson abbia capito la situazione

Mycroft is sure that Watson has understood the situation.

Mycroft obbliga Watson a salire in macchina minacciandolo

Mycroft forces Watson into the car by threatening him.

Watson chiede come fa Mycroft a muovere le videocamere

Watson asks how Mycroft moves the cameras.

Watson indica a Mycroft la posizione delle videocamere

Watson points Mycroft to the location of the cameras.

**#16 In auto con sconosciuta (In car with stranger)**

o/c

Anthea rimane a volto completamente scoperto

Anthea’s remains completely barefaced.

Il cellulare di Anthea è di colore azzurro chiaro

Anthea’s cell phone is light blue in color.

ll dottor Watson ha una luce puntata dritta sugli occhi

Dr. Watson has a light pointed straight at his eyes.

Anthea è vestita con un completo di colore nero

Anthea is dressed in a black suit

s

Anthea è seduta alla sinistra del dottor John Watson

Anthea is sitting to the left of Dr. John Watson.

Il bastone del dottor Watson è sulle gambe di Anthea

Dr. Watson’s cane is on Anthea’s legs.

Il dottor John Watson e Anthea sono ripresi frontalmente

Dr. John Watson and Anthea are filmed frontally.

Il cellulare di Anthea è posizionato sul sedile dell'auto

Anthea’s cell phone is placed on the car seat.

t

Anthea inizia a parlare poi è il turno del dottor Watson

Anthea starts talking then it’s Dr. Watson’s turn.

Watson parla con Anthea mentre l'auto è in movimento

Watson talks to Anthea while the car is in motion.

John Watson si guarda intorno dai finestrini mentre parla

John Watson looks around through the windows as he speaks

Watson sta in silenzio poi Anthea inizia a scrivere

Watson is silent then Anthea begins to write.

v

Anthea confessa che quello non è il suo vero nome

Anthea confesses that that is not her real name.

Anthea sostiene di non conoscere il nome di Watson

Anthea claims not to know Watson’s name.

John Watson chiede di sapere dove stanno andando

John Watson demands to know where they are going.

Watson si presenta dicendo solo il proprio cognome

Watson introduces himself by saying only his last name.

**#17 Magazzino isolato (Isolated warehouse)**

o/c

Il piccolo taccuino di Mycroft è di colore scuro

Mycroft's little notebook is dark colored.

John Watson ha un tremore continuo alla gamba destra

John Watson has a continuous tremor in his right leg.

Mycroft ha un bastone simile a quello di Watson

Mycroft has a cane similar to Watson's

Mycroft indossa una giacca, un gilet scuro e cravatta

Mycroft wears a jacket, dark vest and tie.

s

Mycroft è appoggiato alla sedia e la tiene di fianco a lui

Mycroft is leaning on the chair and holding it beside him.

Mycroft è posizionato al centro del magazzino

Mycroft is positioned in the center of the warehouse.

Watson e Mycroft sono posizionati uno di fronte all’altro

Watson and Mycroft are positioned facing each other.

Mycroft è rivolto verso il retro dell'automobile

Mycroft is facing the back of the car.

t

L'automobile si ferma nel magazzino poi arriva Mycroft

The car stops in the warehouse then Mycroft arrives.

Mycroft guarda l'agenda poi controlla la mano di Watson

Mycroft looks at the diary then checks Watson's hand.

Watson legge l'sms di Sherlock poi incontra Mycroft

Watson reads Sherlock’s text message then meets Mycroft.

Watson scende dall'auto poi inizia il dialogo con Mycroft

Watson gets out of the car then begins dialogue with Mycroft.

v

Mycroft dice a Watson che la sua è nostalgia della guerra

Mycroft tells Watson that his is nostalgia for the war.

Mycroft dice che la psicologa aveva ragione su tutto

Mycroft says the psychologist was right about everything.

Mycroft dice a Watson di scegliere da che parte stare

Mycroft tells Watson to pick a side.

Watson dice che Mycroft gli sembra molto spaventoso

Watson says Mycroft looks very scary to him.

**#18 Watson saluta Anthea (Watson says goodbye to Anthea)**

o/c

L'auto di Anthea ha dei lampeggianti accesi sul tetto

Anthea’s car has flashing lights on the roof.

L'automobile nera di Anthea ha i fari anteriori accesi

Anthea's black car has its headlights on.

Il dottor John Watson è immobilizzato da una stretta cintura

Doctor John Watson is restrained by a tight belt.

L'automobile nera di Anthea ha tutti i finestrini chiusi

Anthea’s black car has all the windows closed.

s

Anthea è posizionata esattamente accanto alla porta di casa

Anthea is positioned exactly next to the front door.

John Watson e Anthea sono entrambi seduti nei posti dietro

John Watson and Anthea are both sitting in the back seats

L'auto è dal lato opposto della strada rispetto alla casa

The car is on the opposite side of the road from the house.

L'auto è sulla sinistra e il palazzo sulla destra della scena

The car is on the left and the building on the right of the scene.

t

L'auto viene parcheggiata poi Watson parla con Anthea

The car is parked and then Watson talks to Anthea.

Watson parla con Anthea prima che lei inizi a scrivere

Watson talks to Anthea before she starts writing.

Il dottor Watson bussa alla porta poi la macchina riparte

Doctor Watson knocks on the door then the car drives off again

Watson scende dall'auto mentre Anthea scrive al cellulare

Watson gets out of the car while Anthea text on her cell phone.

v

Anthea dice a John Watson che lei ha già riferito a Mycroft

Anthea tells John Watson that she has already reported to Mycroft.

Watson chiede ad Anthea di non riferire al capo una cosa

Watson asks Anthea not to tell the boss something

Anthea dice a Watson che lei non ha mai tempo libero

Anthea tells Watson that she never has free time.

Il dottor Watson scende dalla automobile senza salutare

Doctor Watson gets out of the car without saying goodbye.

**#19 Sherlock e Watson nell'appartamento (Sherlock and Watson in the apartment)**

o/c

I cerotti alla nicotina di Sherlock Holmes sono una trentina

Sherlock Holmes' nicotine patches number about thirty.

Sherlock tiene le mani giunte come in segno di preghiera

Sherlock holds his hands together as if in prayer.

La valigia aperta è posizionata sopra una sedia

The suitcase is closed with a huge steel padlock

Si vede Sherlock che indossa la camicia senza giacca

Sherlock can be seen wearing the shirt without a jacket.

s

I cerotti alla nicotina sono sparsi su tutto il pavimento

Nicotine patches are scattered all over the floor.

Il divano con Sherlock si trova accanto alla parete

The sofa with Sherlock is located next to the wall.

Sherlock Holmes è sdraiato sul divano a pancia all'ingiù

Sherlock Holmes is lying on the sofa on his stomach

La valigia aperta è posizionata sopra una sedia

The open suitcase is placed on top of a chair.

t

Il flashback del ritrovamento della valigia è prima dei cerotti

The flashback of finding the suitcase is before the patches.

Si vede Sherlock Holmes sul divano alla fine della scena

Sherlock Holmes is on the sofa near the end of the scene.

Watson entra in casa poi Sherlock prende la valigia

Watson gets into the house then Sherlock picks up the suitcase.

Si vedono prima i cerotti di Sherlock poi la valigia rosa

Sherlock’s patches are seen first then the pink suitcase.

v

Sherlock chiede a John Watson di prestargli il cellulare

Sherlock asks John Watson to lend him his cell phone.

Sherlock dice che la vittima lasciava il cellulare a casa

Sherlock says victim left cell phone at home.

Sherlock dice che di solito la gente non sospetta di lui

Sherlock says that people don't usually suspect him.

Watson racconta a Sherlock di avere incontrato Mycroft

Watson tells Sherlock that he met Mycroft.

**#20 Passeggiata verso il ristorante (Walk to the restaurant)**

o/c

Il dottor Watson indossa un berretto schiacciato sulla fronte

Doctor Watson wears a cap crushed on his forehead.

Sherlock Holmes e dottor Watson hanno i capelli bagnati

Sherlock Holmes and Dr. Watson's hair is wet.

Si vedono delle automobili che sono principalmente scure

Cars are shown mainly dark-colored.

Si vedono i pantaloni di John Watson che sono di colore scuro

John Watson’s trousers are shown dark in color.

s

Sherlock Holmes e il dottor John Watson sono ripresi di profilo

Sherlock Holmes and Dr. John Watson are filmed in profile.

Sherlock Holmes e John Watson camminano uno di fianco all'altro

Sherlock Holmes and John Watson walk side by side.

Sherlock Holmes e John Watson sono ripresi dall'alto verso il basso

Sherlock Holmes and John Watson are shot from top to bottom

Sherlock Holmes e Watson camminano entrambi sul marciapiede

Sherlock Holmes and Watson both walk on the sidewalk.

t

Passano delle auto mentre i due camminano sul marciapiede

Cars pass by as the two walk on the sidewalk.

Sherlock Holmes e Watson parlano mentre camminano insieme

Sherlock Holmes and Watson talk as they walk together.

I due attraversano un incrocio trafficato alla fine della scena

The two walk through a busy intersection at the end of the scene.

Sherlock Holmes fa una giravolta mentre attraversa l'incrocio

Sherlock Holmes makes a turn as he crosses intersection.

v

Sherlock dice che l'assassino riesce a passare inosservato ovunque

Sherlock says the killer can go unnoticed anywhere.

Sherlock Holmes dice che le vittime si allontanavano e poi sparivano

Sherlock Holmes says that the victims walked away and then disappeared

Sherlock dice che quelli intelligenti non vogliono farsi catturare

Sherlock says the smart ones do not want to get caught.

Sherlock Holmes si chiede chi caccia in mezzo a una folla

Sherlock Holmes wonders who he hunts in a crowd.

**#21 Al ristorante (At the restaurant)**

o/c

Apparentemente il ristoratore avrà non più di 30 anni d'età

Apparently the restaurateur is no more than 30 years og age.

Il proprietario del ristorante Angelo porta anche la barba

Restaurant owner Angelo also wears a beard.

Il ristoratore appare come un uomo dal fisico esile

The restaurateur appears as a man with a tiny physique

Il ristoratore porta i capelli lunghi con la coda

The restaurant owner wears his long hair in a ponytail.

s

Il ristoratore si trova sul lato sinistro dello schermo

The restaurateur is located on the left side of the screen.

La finestra è posizionata alle spalle di John Watson

The window is positioned behind John Watson's back.

Il menu del ristorante è già posizionato sul tavolo

The restaurant menu is already placed on the table

Sherlock e Watson siedono l'uno di fronte all'altro

Sherlock and Watson sit across from each other.

t

Il dottor John Watson guarda un menu poi si siede

Dr. John Watson looks at a menu and then sits down.

Sherlock entra nel ristorante poi si vede il ristoratore

Sherlock enters the restaurant and then the restaurateur is seen.

Il ristoratore accoglie Sherlock e Watson poi i due si siedono

The restaurateur greets Sherlock and Watson then the two sit down.

Watson parla mentre Sherlock guarda fuori dalla finestra

Watson talks while Sherlock looks out the window.

v

Il dottor Watson dice che ciascuno di noi ha un acerrimo nemico

Dr. Watson says each of us has a bitter enemy.

Sherlock dice a Watson di avere visto un taxi sospetto

Sherlock tells Watson he has seen a suspicious cab.

Sherlock Holmes sostiene di essere sposato col proprio lavoro

Sherlock Holmes claims to be married to his work

Sherlock dice che il ristoratore è finito in carcere per omicidio

Sherlock says the restaurateur went to jail for murder.

**#22 Un taxi sospetto (Suspicious cab)**

o/c

Il dottor John Watson è ripreso dal basso verso l'alto

Doctor Watson is filmed from the bottom up.

John Watson è vestito con un maglioncino chiaro

John Watson is dressed in a light-colored sweater.

John Watson si nasconde il volto con una mano

John Watson hides his face with one hand.

Sherlock Holmes indossa una giacca e la camicia

Sherlock Holmes is wearing a jacket and shirt

s

Il taxi con il turista americano è ripreso dall'alto

The cab with the American tourist is filmed from above.

Il taxi con il turista è dall'altro lato della strada

The cab with the tourist is on the other side of the road.

Sherlock e Watson sono ripresi dal basso in alto

Sherlock and Watson are filmed from bottom to top

Il taxi fermo è alla destra di Sherlock al ristorante

The stationary cab is to Sherlock's right at the restaurant.

t

John Watson esce dal ristorante prima di Sherlock

John Watson exits the restaurant before Sherlock.

Dopo che i due sono usciti si vede il bastone di Watson

After the two have left Watson's cane appears.

Sherlock fa notare il taxi e Watson si gira a guardare

Sherlock points out the cab and Watson turns to look.

Watson si alza in piedi poi Sherlock indica il taxi

Watson stands up and then Sherlock points to the cab.

v

John Watson dice che Sherlock sta fissando il taxi

John Watson says Sherlock is staring at the cab.

Sherlock dice che si è fermato un taxi ma non è sceso nessuno

Sherlock says a cab stopped but no one got out.

Sherlock dice che John Watson è veramente furbo

Sherlock says John Watson is really sly

Sherlock invita il dottor Watson a fissare il taxi

Sherlock inivtes Dr. Watson to stare at the cab.

**#23 Inseguimento (Chase)**

o/c

Il taxi che colpisce Sherlock si muoveva con i fari spenti

The cab that hit Sherlock was moving with its headlights off.

Le due linee della mappa sono intersecate in più di un punto

The two lines in the map are intersected at more than one point.

Le due linee in movimento sulla mappa sono di diverso colore

The two shifting lines on the map are different colors.

Sherlock Holmes usa un navigatore gps per orientarsi meglio

Sherlock Holmes uses a gps navigator to better orient himself.

s

Si vedono le scale a chiocciola che sono riprese dal basso

The spiral staircase are seen filmed from below.

Un'auto frena ed investe Sherlock davanti al ristorante

A car brakes and runs over Sherlock in front of the restaurant.

Il dottor John Watson è davanti a Sherlock Holmes

Dr. John Watson stands in front of Sherlock Holmes.

Watson è sempre ripreso dall'alto durante la corsa

Watson is always filmed from above during the race.

t

Il taxi parte poi Sherlock Holmes inizia a corrergli dietro

The cab leaves then Sherlock Holmes starts running after it.

John Watson inizia a correre prima di Sherlock Holmes

John Watson starts running before Sherlock Holmes.

L'auto investe Sherlock mentre lui immagina il percorso

Car hits Sherlock while he imagines the route.

Si vede in continua alternanza la mappa e i due che corrono

A continuous alternation of the map and the two men running is seen.

v

Il dottor John Watson urla a Sherlock Holmes di seguirlo

Doctor Watson yells at Sherlock Holmes to follow him.

Sherlock incoraggia Watson e dà indicazioni sul percorso

Sherlock encourages Watson and gives directions on the route.

Sherlock urla a John Watson di dividere le loro direzioni

Sherlock yells at John Watson to divide their directions.

Watson dice di aver preso il numero di targa del taxi

Watson says he got the cab's license plate number.

**#24 Dialogo con il cliente (Dialogue with customer)**

o/c

Il turista americano ha i capelli di colore scuro

The American tourist has dark-colored hair.

Il turista americano indossa un orecchino d'oro

The American tourist wears a gold earring.

Il turista americano ha la barba completamente rasata

The American tourist has a completely shaved beard

Il turista americano indossa una cravatta rossa

The American tourist wears a red tie.

s

Il turista è nel posto lontano dallo sportello aperto

The tourist is located away from the car’s open door.

Il turista è più vicino a Sherlock rispetto a Watson

The turist is closer to Sherlock than Watson.

John Watson è di fronte allo sportello dell'autista

John Watson is in front of the driver's door.

Sherlock e Watson sono alla sinistra del turista

Sherlock and Watson are to the left of the tourist.

t

Sherlock mostra il distintivo poi apre lo sportello

Sherlock shows her badge then opens the door.

Sherlock parla con il turista prima di John Watson

Sherlock talks to the tourist before John Watson.

Watson chiude lo sportello poi si vede il bagaglio

Watson closes the door then the luggage is seen.

Sherlock si allontana poi Watson chiude lo sportello

Sherlock walks away then Watson closes the door

v

Il turista chiede se Sherlock e Watson sono della polizia

The tourist asks if Sherlock and Watson are from the police.

Watson dice al turista di chiamarli in caso di problemi

Watson tells the tourist to call them if there is any problem.

Sherlock dice che il turista ha la pelle molto chiara

Sherlock says the tourist has very pale skin.

Sherlock Holmes dice che il turista è già stato a Londra

Sherlock Holmes says the tourist has already been to London.
